# Sticker Valence Governs Chain Collapse and Liquid‐Liquid Phase Separation in Peptide‐Inspired Associative Polymers

**DOI:** 10.1002/advs.77086

**Published:** 2026-08-18

**Authors:** Seung‐Hwan Oh, Jinhoon Lee, Seunghyun Lee, Raehyun Jung, Sangyeop Lee, Su‐Mi Hur, Dong Woog Lee, Soo‐Hyung Choi

**Affiliations:** ^1^ Department of Chemical Engineering Hongik University Seoul Republic of Korea; ^2^ School of Energy & Chemical Engineering Ulsan National Institute of Science and Technology (UNIST) Ulsan Republic of Korea; ^3^ Department of Energy Science and Engineering Daegu Gyeongbuk Institute of Science and Technology (DGIST) Daegu Republic of Korea; ^4^ School of Polymer Science and Engineering Chonnam National University Gwangju Republic of Korea

**Keywords:** associative polymer, liquid‐liquid phase separation, peptidomimetics, self‐assembly, stickers‐and‐spacers framework

## Abstract

Biomolecular condensates, including membraneless organelles, form through liquid‐liquid phase separation (LLPS) driven by sequence‐specific associations within intrinsically disordered protein regions. Understanding how sticker valence governs LLPS is essential for predicting pathological aggregation and designing programmable biomaterials. Among associative amino acid motifs, cationic arginine (Arg) is a key regulator of biological LLPS, yet the molecular basis of its distinct phase behavior remains incompletely understood. Here, we investigate peptide‐inspired heteropolymers composed of associative Arg‐like guanidinium stickers and lysine‐like ammonium spacers. The interchain sticker–sticker associations lead to LLPS, and sticker valence governs the phase separation behavior. Prior to macroscopic phase separation, enhanced sticker associations induce a continuous coil‐to‐globule transition even under overall good‐solvent conditions, thereby facilitating LLPS. At interfaces, elevated salt counterintuitively reduces inter‐surface cohesion despite stronger sticker associations, because preferential intra‐surface network formation depletes free stickers available for inter‐surface binding; this cohesion can be transiently restored by mechanical stimulation. These results establish a molecular framework linking sticker valence, chain collapse, phase separation, and interfacial mechanics in associative polymers, and identify peptide‐inspired sticker–spacer heteropolymers as a versatile platform for programmable condensates and responsive soft materials.

## Graphical abstract

Peptide‐inspired associative polymers with arginine‐like guanidinium stickers and lysine‐like ammonium spacers undergo chain collapse and liquid‐liquid phase separation induced by reversible sticker associations. Sticker valence and tunable association strength jointly control the phase behavior, chain conformation, and interfacial cohesion of these sticker–spacer polymers in aqueous media, thereby establishing a molecular framework for biomolecular condensation and programmable soft materials.

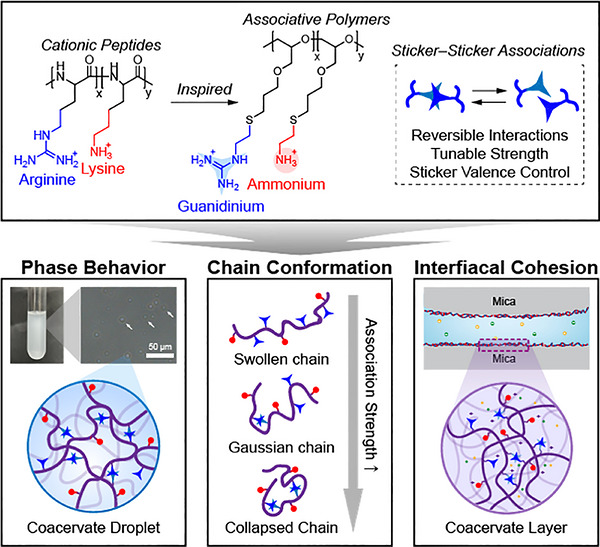

## 1 Introduction

Membraneless organelles are dynamic biomolecular condensates that lack a surrounding lipid membrane and organize intracellular biochemical space in a spatiotemporally regulated manner [1−3]. These condensates arise from liquid‐liquid phase separation (LLPS) of proteins and nucleic acids and have emerged as a fundamental mechanism for compartmentalizing and regulating biochemical reactions across multiple length scales [3−5]. While LLPS plays essential roles in diverse cellular processes, including genome organization, immune signaling, synaptic function, and transcription [6−9], dysregulated condensation can also promote pathological protein aggregation associated with neurodegenerative diseases and cancer [10−14]. The understanding of biological LLPS can be traced back to the seminal work of Bungenberg de Jong and Kruyt on coacervation [15], which describes spontaneous phase separation into two coexisting liquid phases: a biomolecule‐rich condensate (i.e., coacervate) and a dilute supernatant [16, 17]. Building on this foundational framework, recent studies have highlighted that the formation and stability of these condensates are governed by the cooperative interplay of multivalent interactions, including electrostatic, *π*–*π*, cation–*π*, and hydrophobic interactions [18−25]. In particular, the molecular grammar of interaction motifs enriched in charged, polar, and aromatic residues dictates the sequence specificity and phase behavior of LLPS‐prone proteins [26−28]. Elucidating these molecular principles has not only advanced our understanding of intracellular organization, but has also inspired the development of synthetic systems that emulate LLPS. Such biomimetic condensates provide promising platforms for engineering dynamic, stimuli‐responsive soft materials with programmable phase behavior. Establishing a molecular framework for such systems therefore requires concepts that connect sequence architecture to associative phase behavior.

Phase separation is a foundational concept in polymer science [29]. For decades, Flory−Huggins theory has provided a theoretical framework for describing the spontaneous demixing of homopolymeric systems in terms of enthalpic and entropic contributions [30−32]. In contrast to homopolymers, however, most biomolecules are heteropolymers with sequence‐defined structures and chemically diverse motifs. To describe the phase behavior of such systems, recent studies have increasingly adopted the associative polymer framework, often formulated as the stickers‐and‐spacers model, to rationalize the sequence‐encoded mechanisms underlying biological LLPS [33−35]. In this model, the heterogeneous architecture of a biopolymer is reduced to two functional elements: “stickers”, which represent associative motifs capable of reversible intra‐ and inter‐molecular interactions, and “spacers”, which separate these stickers along the polymer backbone [36−38]. Associative phase separation is driven primarily by attractive sticker–sticker interactions, giving rise to coexisting polymer‐rich, and polymer‐lean phases. Importantly, sticker concentration, interaction strength, and valence, that is, the number of stickers per chain, collectively govern the extent of phase separation as well as the dynamics and material properties of the condensed phase [34, 37]. The stickers‐and‐spacers framework has successfully recapitulated and predicted key experimental observations of LLPS in biological systems [39−44]. These advances suggest that synthetic sticker−spacer polymers with peptide‐like interaction motifs and reduced compositional complexity can serve as powerful model systems for elucidating associative phase behavior and as promising platforms for bio‐inspired functional materials.

Among candidate associative motifs, arginine (Arg), which bears a cationic guanidinium (G) group, has emerged as a particularly promising sticker unit for peptide‐inspired sticker−spacer polymers. Arg‐rich motifs are widely recognized as potent drivers and regulators of biomolecular LLPS, largely because they form strong electrostatic interactions with anionic biomolecules such as DNA and RNA [45−47]. Notably, although both Arg and lysine (Lys), which bears a cationic ammonium (A) group, carry a single positive charge under physiological conditions, Arg consistently exhibits a substantially higher propensity to promote LLPS than Lys [45−52]. This distinct behavior is generally attributed to the amphiphilic and quasi‐aromatic character of the guanidinium group, which is sp^2^‐hybridized, planar, and electronically delocalized over three amine substituents. These molecular features enable *π*‐mediated associations, including homotypic interactions between like‐charged Arg residues in water [21, 53−59]. As a consequence, Arg‐rich sequences can form dynamic yet cohesive liquid‐like networks that are highly sensitive to their environment. Indeed, Arg‐rich intrinsically disordered proteins such as protamine have been shown to undergo phase separation even at physiological to high ionic strengths without requiring oppositely charged macromolecular partners [60, 61]. This behavior resembles previously reported self‐ or simple coacervation in like‐charged polypeptides, where non‐electrostatic driving forces, such as cation–*π* interactions, induce phase separation despite Coulombic repulsion [23, 62]. Inspired by these observations, we previously demonstrated that homopolymeric polyguanidinium chains, composed entirely of G stickers, undergo salt‐dependent LLPS through tunable *π*–*π* interactions between guanidinium groups [63, 64]. This phase behavior closely resembles that of hydrophobic polypeptides [24, 65]. However, unlike hydrophobic interactions, which are often difficult to quantify and systematically control, guanidinium‐mediated association provides a chemically tunable interaction motif whose strength can be modulated by the ionic environment in water [66, 67].

**FIGURE 1:**
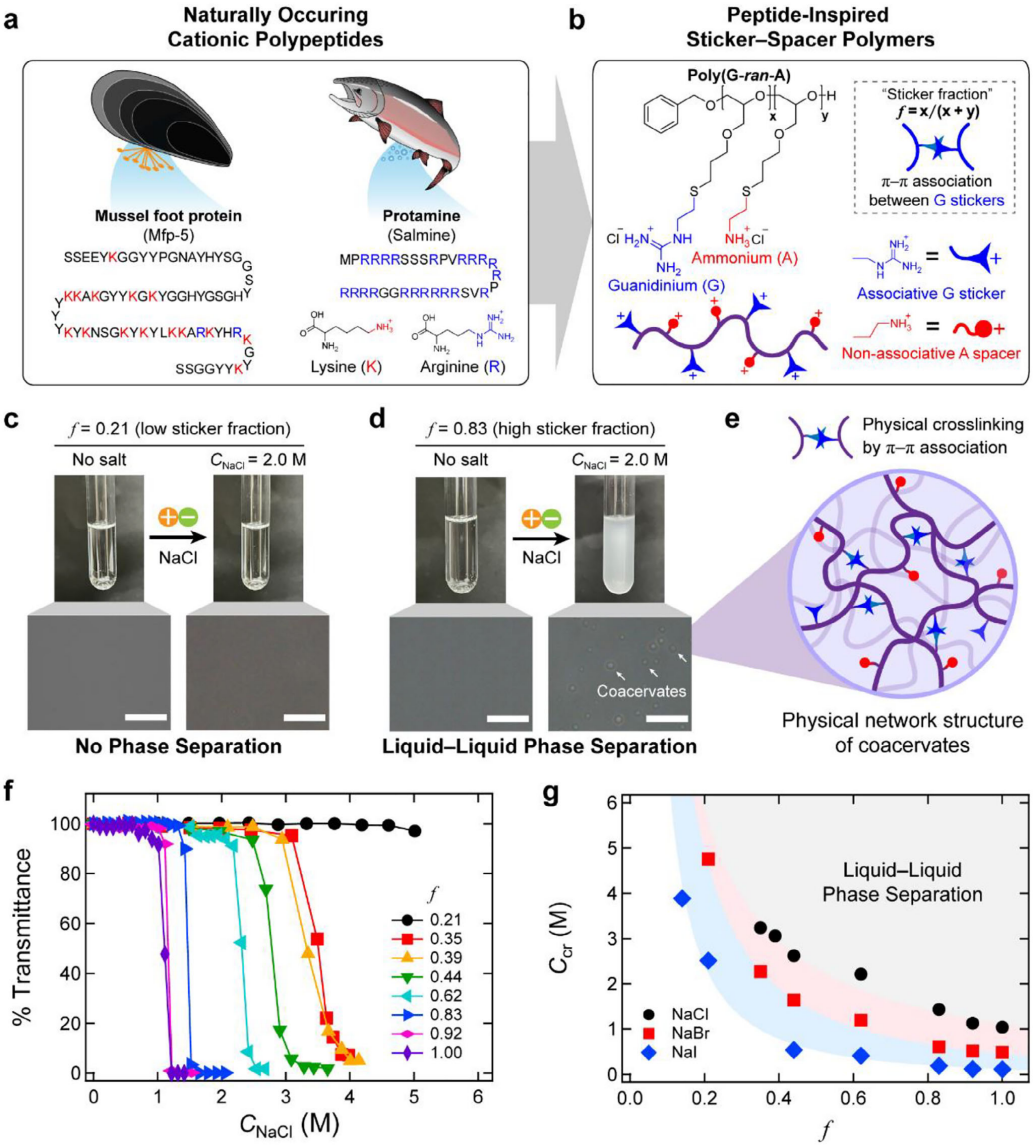
(a) Schematic illustration of naturally occurring cationic polypeptides containing arginine (Arg) residues. (b) Molecular design of peptide‐inspired sticker−spacer polymers composed of guanidinium (G) stickers and ammonium (A) spacers. Photographs and optical microscopic images of 0.1 wt.% poly(G‐*ran*‐A) aqueous solutions with (c) low sticker fraction (*f* = 0.21) and (d) high sticker fraction (*f* = 0.83) before and after NaCl addition. Micron‐sized coacervate droplets are indicated by arrows; scale bars, 50 µm. (e) Schematic illustration of the physical network structure formed through associative *π*–mediated interactions between G stickers. (f) Optical transmittance of 0.1 wt.% poly(G‐*ran*‐A) solutions as a function of NaCl salt concentration (*C*_NaCl_) at 23°C over a range of *f*. (g) Critical salt concentration (*C*_cr_) as a function of *f* for NaCl (black), NaBr (red), and NaI (blue) at 23°C. Shaded regions denote the biphasic phase‐separated regimes.

Despite these advances, a quantitative molecular framework connecting sticker valence, chain conformation, and phase separation in peptide‐inspired heteropolymers remains elusive. Here, we show that both sticker valence and sticker–sticker association strength can be systematically tuned to control the LLPS behavior of peptide‐inspired sticker−spacer heteropolymers. To mimic the architecture of naturally occurring cationic polypeptides such as mussel foot proteins and protamine (Figure 1a) [68, 69], we designed random copolymers composed of Gstickers and Aspacers, denoted (poly(G‐*ran*‐A)), which emulate the Arg and Lys residues, respectively (Figure 1b). Using optical transmittance, small‐angle neutron scattering (SANS), static light scattering (SLS), coarse‐grained simulations, and surface force apparatus (SFA) measurements, we uncover how sticker valence and ionic strength jointly regulate chain conformation, phase separation, and pairwise interactions in peptide‐inspired sticker–spacer heteropolymers. These results identify poly(G‐*ran*‐A) as a tunable bio‐inspired platform for programmable LLPS in aqueous media through the combined control of sequence composition and ionic environment.

## 2 Results and Discussion

### 2.1 Sticker‐Valence‐ and Salt‐Dependent Liquid‐Liquid Phase Separation

Poly(allyl glycidyl ether) (PAGE), synthesized by living anionic ring‐opening polymerization, was selected as a modular scaffold. Its hydrophilic poly(ethylene glycol) (PEG)‐like polyether backbone minimizes hydrophobic contributions to phase behavior, while its pendant allyl groups enable facile post‐polymerization modification [70]. To mimic the cationic residues of natural polypeptides, lysine (Lys)‐like ammonium (A), and arginine (Arg)‐like guanidinium (G) groups were subsequently introduced onto the PAGE main chain by thiol‐ene chemistry and guanylation, respectively (Figure S1) [71, 72]. A series of cationic random copolymers, poly(G‐*ran*‐A), was synthesized with identical number‐average degree of polymerization (*N*_n_ = 130) and narrow dispersity (*Đ* = 1.09) (Figures S2 and S3). The sticker fraction (*f*), defined as the molar fraction of G units, was systematically varied from 0.00 to 1.00 by substituting A groups with G while maintaining the net positive charge per chain (Figures S4 and S5) [72].

Consistent with our previous observation that homopolymeric polyguanidinium (*f* = 1.00) undergoes salt‐induced LLPS through associative *π*–mediated interaction between G stickers [63, 64], we hypothesized that tuning *f* would modulate the LLPS propensity of poly(G‐*ran*‐A). Indeed, LLPS was strongly dependent on sticker valence. At 0.1 wt.% polymer concentration, poly(G‐*ran*‐A) with low G content (*f* = 0.21) remained optically transparent up to 2.0 m NaCl (Figure 1c), whereas the polymer with *f* = 0.83 exhibited pronounced turbidity under identical conditions (Figure 1d). Optical microscopy revealed micron‐sized droplets in the turbid samples, confirming coacervate formation. Because A groups do not participate in associative interactions, they act as non‐interacting spacers that dilute the effective valence of G‐mediated interactions. Given the identical chain lengths across the series, these data identify sticker valence as a key determinant of reversible *π*–mediated association and LLPS in poly(G‐*ran*‐A) solutions (Figure 1e).

To quantify this valence dependence, we measured the optical transmittance of 0.1 wt.% poly(G‐*ran*‐A) aqueous solutions as a function of NaCl concentration (*C*_NaCl_) over a broad range of *f* values (Figure 1f). LLPS onset was marked by a sharp decrease in transmittance, indicating the formation of a biphasic system. We defined the critical salt concentration (*C*_cr_) as the NaCl concentration where the transmittance reached 80% and plotted it against *f* (Figure 1g). *C*_cr_ decreased from 3.2 to 1.0 m as f increased from 0.35 to 1.00, whereas no LLPS was observed below *f* = 0.35 even at saturated NaCl concentrations. Thus, both sticker valence and ionic strength govern the probability of reversible sticker−sticker association and thereby control LLPS in this bio‐inspired system.

This trend can be rationalized within the associative polymer framework [37] in which a chain of length *N* contains *N*_st_ stickers separated on average by *l* = *N*/*N*_st_ = *f^–^* ^1^. In this model, the probability *p* of sticker−sticker association is described by

(1) $$\frac{\;p\;}{{\left(1-p\right)}^{2}}=\frac{c_{m}\;}{l}\lambda_{att}$$

where *c*_m_ is the monomer concentration and *λ*_att_ is the association strength, defined as *λ*_att_ = *ν*_b_ exp(*E*_a_/*k*_B_ *T*) with *ν*_b_ the volume of an associative bond, *E*_a_ the interaction energy, *k*_B_ the Boltzmann constant, and *T* the absolute temperature. Although this treatment is typically applied to semi‐dilute systems, the gelation threshold *p*_c_ can reasonably serve as a proxy for LLPS in our dilute regime because network formation develops within the dense coacervate phase [33, 39]. At constant *c*_m_, increasing ionic strength enhances *λ*_att_, thereby promoting network connectivity. Because *p*_c_ decreases with increasing sticker valence (*p*_c_ = 1/(*N*_st_ – 1)), polymers with lower *f* require stronger attractive interactions, that is, higher ionic strength, to reach the LLPS boundary. This picture is fully consistent with the experimentally observed inverse relationship between *C*_cr_ and *f*.

The strong salt dependence further suggested that ion identity should also modulate LLPS. Indeed, substituting NaCl with NaBr or NaI enhanced phase separation, enabling LLPS even at *f* = 0.14 and 0.21 (Figure 1g and Figure S6). The critical salt concentrations followed the order NaI < NaBr < NaCl, consistent with the inverse Hofmeister series for halide anions [73]. This behavior reflects the greater ability of more polarizable, weakly hydrated anions to screen electrostatic repulsion, and promote associative interactions [63, 74, 75]. It is noted that LLPS of poly(G‐*ran*‐A) solutions with *f* = 1 is also triggered by multivalent salts such as sodium sulfate (Na_2_SO_4_) and trisodium citrate (Na_3_C_6_H_5_O_7_) at extremely low salt concentrations (Figure S7a), whereas solutions without stickers (*f* = 0) remain transparent up to the solubility limits of the multivalent salts (Figure S7b). This finding suggests that LLPS of poly(G‐*ran*‐A) is primarily attributed to G sticker interactions, whereas the intermolecular salt bridges between the cationic sticker/spacer units and multivalent anions alone are insufficient to induce phase separation [63]. Collectively, these results demonstrate that association‐induced LLPS is governed jointly by sticker valence and salt chemistry, highlighting the interplay between molecular architecture and solvent environment.

Next, we investigated the effect of molecular association inhibitors on LLPS using 1,6‐hexanediol and guanidinium chloride (GdmCl) as hydrophobic and homotypic *π*–mediated interaction disruptors, respectively (Figure S8). The addition of the hydrophobic interaction disruptor slightly shifted the LLPS boundary of poly(G‐*ran*‐A) solutions with *f* = 1.00 to a higher *C*_NaCl_ (Figure S8a), indicating that the hydrophobicity of amphiphilic G stickers partially contributes to phase separation [47]. However, the LLPS was completely suppressed upon addition of GdmCl (Figure S8b), consistent with competitive disruption of interpolymer stacking by free guanidinium ions. Furthermore, adding a PEG molecular crowder had only a marginal effect on LLPS, although PEG has been reported to facilitate complex coacervation through an entropy gain from water release upon dehydration (Figure S8a) [76]. These observations are consistent with our previous report [64], supporting that LLPS of G‐containing associative polymers is enthalpically driven by attractive *π*–mediated interactions between G stickers rather than by entropic contributions, including hydration.

### 2.2 Sticker‐Valence‐ and Salt‐Dependent Chain Collapse

To determine how sticker valence influences chain conformation prior to macroscopic phase separation, we next examined the salt‐dependent coil‐to‐globule transition (CGT). In associative polymer solutions, monomer–monomer interactions are modulated by reversible sticker association, while monomer–solvent interactions remain largely unchanged [37]. As salt concentration increases, enhanced sticker–sticker association drives chain collapse from an extended coil to a compact globule [77]. This CGT is characterized by a decrease in the radius of gyration (*R*_g_) and the Flory exponent (*ν*), where *ν* = 0.6, 0.5, and 0.33 correspond to good, ideal, and poor solvent conditions, respectively [78]. Because sticker association effectively lowers the apparent solvent quality, the experimentally determined effective Flory exponent (*ν*_eff_) and *R*_g_ provide quantitative measures of conformational changes induced by reversible sticker–sticker interactions.

**FIGURE 2:**
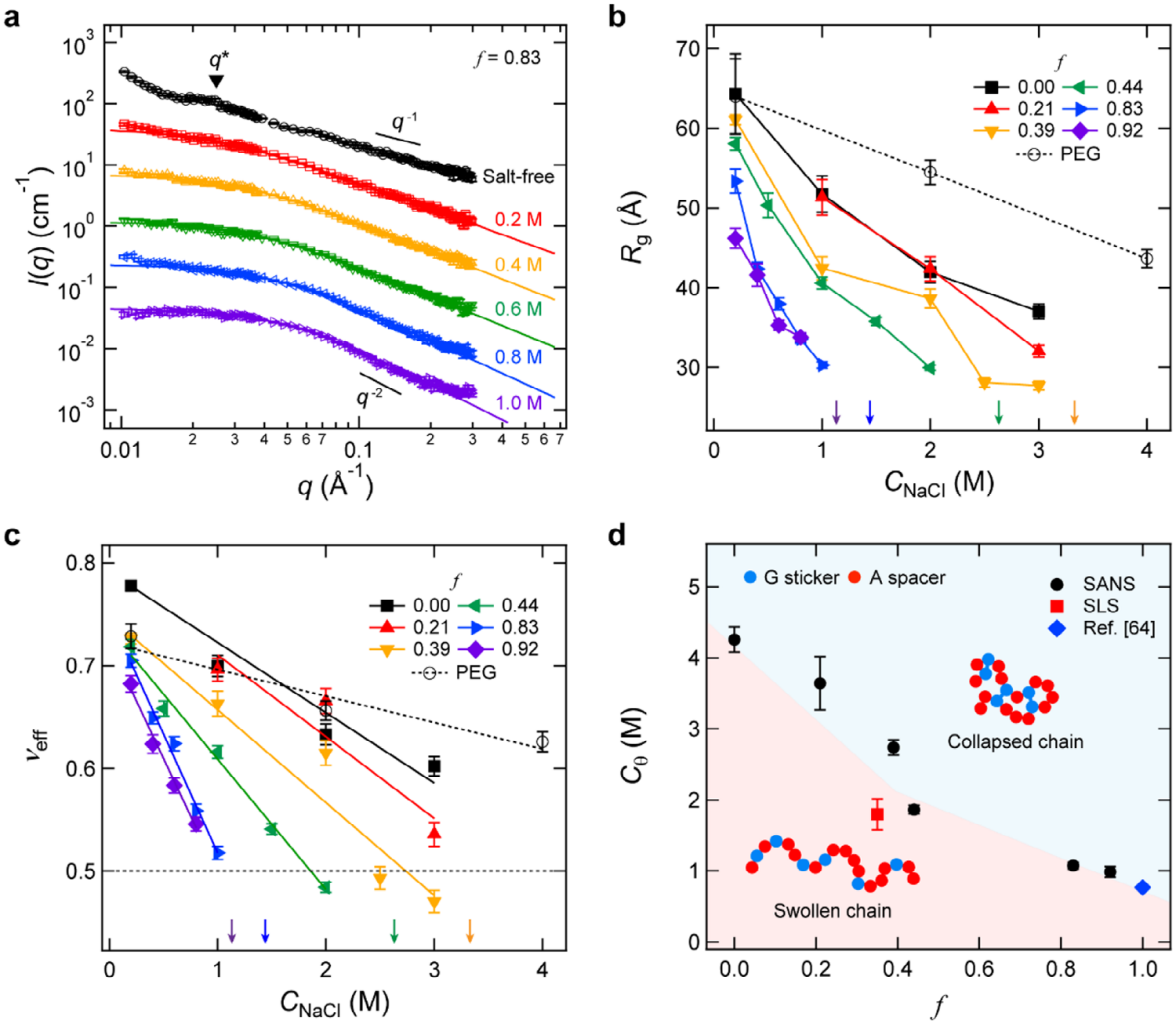
(a) SANS profiles for 0.1 wt.% poly(G‐*ran*‐A) solutions with *f* = 0.83 at various *C*_NaCl_. Solid lines represent fits to *P*(*q*) (Equation 2). (b) Radius of gyration (*R*_g_) and (c) Effective Flory exponent (*ν*_eff_) of poly(G‐*ran*‐A) chains at different f as a function of *C*_NaCl_. Arrows indicate *C*_cr_. In panel c, solid, and dashed lines represent linear fit used to determine *C*_θ_ at *ν*_eff_ = 0.5. (d) Experimentally determined *C*_θ_ as a function of *f*. Circle, square and diamond symbols correspond to values obtained from SANS, SLS, and ref. [64], respectively. Red and blue shaded regions denote swollen and collapsed chain conformations, respectively.

To probe CGT behavior, small‐angle neutron scattering (SANS) was performed on 0.1 wt.% poly(G‐*ran*‐A) solutions with varying sticker fractions (*f* = 0.00, 0.21, 0.39, 0.44, 0.83, and 0.92) over a range of *C*_NaCl_ values (Figure 2a). All SANS profiles were collected from homogeneous single‐phase solutions below *C*_cr_ (Figure S9). In the absence of added salt, the scattering intensity *I*(*q*) exhibited a *q^–^* ^1^ dependence in the high‐*q* regime (*q* > 0.04 Å^−1^), consistent with rod‐like chain conformations. A correlation peak at *q* ^*^ ≈ 0.022 Å*^–^* ^1^ was observed for all *f*, corresponding to a correlation length of *ξ* = 2*π*/*q* ^*^ ≈ 290 Å (Figure S10). Given the contour length of PEG (∼ 190 Å for *N* = 130) [79], this result indicates that the highly stretched poly(G‐*ran*‐A) chains remain electrostatically correlated even in dilute solution, despite the absence of topological overlap [80, 81].

With increasing *C*_NaCl_, the high‐*q* slope of *I*(*q*) gradually shifted from *q^–^* ^1^ to *q^–^* ^2^, and the correlation peak disappeared, indicating a transition toward Gaussian chain statistics as electrostatic repulsion was screened. To quantify chain dimensions, the SANS profiles were fitted using a form factor for linear chains with excluded‐volume effects [82]. For comparison, PEG homopolymers (*N*_n_ = 114) were analyzed under identical conditions to evaluate backbone‐specific contributions (Figure S9). Before the onset of LLPS (*C*_NaCl_ < *C*_cr_), *R*_g_ of poly(G‐*ran*‐A) decreased monotonically with increasing *C*_NaCl_ and approached ∼30 Å near *C*_cr_ for all *f*, with the rate of decrease strongly dependent on sticker fraction (Figure 2b). For example, at *f* = 0.83, *R*_g_ decreased sharply from 53 to 30 Å as *C*_NaCl_ increased from 0.2 to 1.0 m, indicating pronounced chain collapse driven by sticker–sticker associations. By contrast, chains without stickers (*f* = 0.00) showed a more gradual decrease in *R*_g_, from 64 to 37 Å over 0.2–3.0 m NaCl. PEG homopolymers exhibited a smaller decrease in *R*_g_, from 64 to 43 Å over 0.2–4.0 m NaCl, indicating that reduced backbone solvation contributes to chain collapse but cannot account for the much stronger compaction observed in sticker‐containing polymers.

The *ν*_eff_ extracted from the SANS profiles provided thermodynamic insight into the salt‐dependent conformational behavior of the sticker−spacer polymers (Figure 2c). At low salt concentration (*C*_NaCl_ = 0.2 m), *ν*_eff_ ranged from 0.7 to 0.8 across all *f* values, indicating highly swollen chain conformations. This behavior indicates that electrostatic repulsion between the charged monomers is sufficiently screened, allowing the chains to adopt conformations comparable to those of neutral polymers under good‐solvent conditions [80]. Consistently, the PEG control exhibited *ν*_eff_ ≈ 0.73 at *C*_NaCl_ = 0.2 m, confirming the good‐solvent character of the polyether backbone. With increasing *C*_NaCl_, *ν*_eff_ of poly(G‐*ran*‐A) decreased progressively, with the rate of decrease strongly dependent on sticker fraction (Figure 2c). For *f* = 0.00, *ν*_eff_ decreased only modestly from 0.78 to 0.60 as *C*_NaCl_ increased from 0.2 to 3.0 m. PEG hains showed an even smaller decrease, from 0.73 to 0.63 over 0.2–4.0 m NaCl, indicating that both the polyether backbone and A spacers remain largely solvated under the present conditions. By contrast, at higher sticker valence (*f* = 0.83), *ν*_eff_ decreased rapidly from 0.70 to 0.52 as *C*_NaCl_ increased from 0.2 to 1.0 m, indicating chain collapse toward Gaussian conformation near *C*_cr_. Importantly, for all values of *f*, the LLPS boundary was reached while the backbone and spacers still remained under overall good‐solvent conditions. Although solvated spacer segments are expected to suppress sticker association [33, 37], the *π*‐mediated interactions between G stickers become sufficiently strong at high ionic strength to overcome these excluded‐volume penalties. These findings highlight the role of sticker valence not only in phase separation, but also in governing conformational transitions at the single‐chain level.

To quantify the conformational transition, we defined the CGT boundary as the theta salt concentration (*C*_θ_). Below *C*_θ_, flexible polymer chains in dilute solutions adopt swollen conformations with only minimal sticker−sticker associations. Above *C*_θ_, the chains collapse as attractive interactions between stickers become dominant. At *C*_θ_, repulsive and attractive interactions are balanced, and the chains assume an ideal Gaussian conformation. Accordingly, *C*_θ_ was defined from the SANS measurements as the *C*_NaCl_ at which *ν*_eff_ = 0.5 (Figure 2d). To independently validate this transition point, we performed static light scattering (SLS) measurements on poly(G‐*ran*‐A) solutions with *f* = 0.35 and identified *C*_θ_ as the *C*_NaCl_ at which the effective osmotic second virial coefficient approached zero (Figure S13) [83]. The *C*_θ_ values obtained from these two independent methods agreed well within experimental uncertainty (Figure 2d). Moreover, the *C*_θ_ value extracted from our previous study on homopolymeric polyguanidinium (*f* = 1.00) was quantitatively consistent with the present result (Figure 2d) [64]. As *f* increased from 0.00 to 1.00, the experimentally determined *C*_θ_ decreased markedly from 4.3 to 0.8 m, indicating that higher sticker valence enhances associative interactions and thereby lowers the apparent solvent quality [37]. Collectively, these results reveal a continuous CGT behavior in which *π*‐mediated interactions between G stickers drive a single‐chain collapse transition even under conditions where the polyether backbone and A spacers remain under overall good‐solvent conditions. This conformational transition precedes and promotes salt‐dependent associative phase separation, underscoring the central role of sticker valence and association strength in governing the phase behavior of associative polymers [77].

**FIGURE 3:**
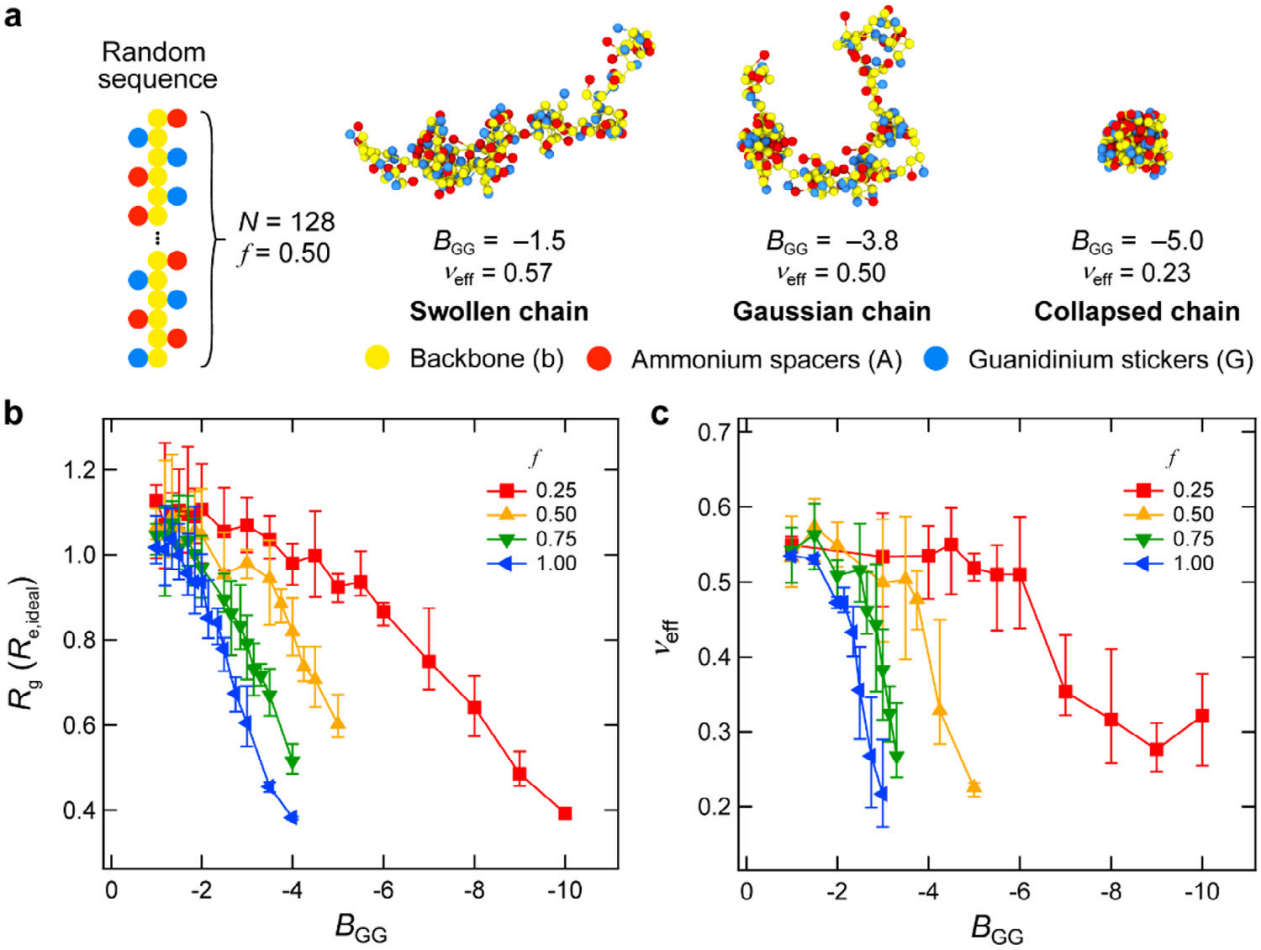
(a) Representative conformations of a single polymer chain (*N* = 128 and *f* = 0.50) under different sticker−sticker interaction strengths, illustrating swollen, Gaussian, and collapsed states. (b) Radius of gyration (*R*_g_), expressed in units of the ideal root ‐ mean‐squared end‐to‐end distance under Gaussian conditions (*R*_e, ideal_), of polymer chains (*N* = 128) with varying *f* as a function of the second virial coefficient for sticker−sticker interactions (*B*_GG_). (c) Effective Flory exponent (*ν*_eff_) extracted from the chain‐size scaling relation $R_{g}\;∼\;N^{\nu_{\mathrm{eff}}}$ as a function of *B*_GG_ for different *f*.

To further substantiate the molecular basis of this association‐induced CGT, we performed coarse‐grained simulations based on a stickers‐and‐spacers model. The model polymers consisted of guanidinium sticker beads (G) and ammonium spacer beads (A) attached to ether‐like backbone beads (b), arranged in a random sequence with sticker fractions *f* = 0.25, 0.50, 0.75, and 1.00 (Figure 3a). Non‐bonded interactions were described within a virial expansion framework as a function of local density fields up to third order (Section 4.2). Pairwise attractive interactions were represented by second virial coefficients for G–G (*B*_GG_), A–A (*B*_AA_), and backbone–backbone (*B*_bb_) pairs, whereas repulsive three‐body interactions were introduced through third virial coefficients *ω*_GGG_, *ω*_AAA_ and *ω*_bbb_, each set to 0.04. To isolate the effect of sticker association, non‐associative interactions were fixed at *B*_AA_ = *B*_bb_ = 0, corresponding to good‐solvent conditions for the spacer and backbone segments, whereas *B*_GG_ was systematically varied to tune sticker–sticker attraction.

Using Monte Carlo (MC) simulations, we computed the *R*_g_ of isolated polymer chains over a degree‐of‐polymerization range of *N* = 32 to 1024, from which the scaling relation $R_{g}\;∼\;N^{\nu_{\mathrm{eff}}}$ was obtained (Figure S14). For a representative case (*N* = 128 and *f* = 0.50), decreasing *B*_GG_ from –1.5 to –5.0 led to a continuous reduction in *ν*_eff_ from 0.57 to 0.23, indicating progressive chain collapse (Figure 3a). The coil‐to‐globule boundary occurred at *B*_GG_ = −3.8, where *ν*_eff_ = 0.5, corresponding to a Gaussian chain conformation. Both *R*_g_ and *ν*_eff_ decreased with increasingly negative *B*_GG_, and the extent of this decrease became more pronounced as *f* increased (Figure 3b,c). This behavior is in good agreement with the experimental SANS results, confirming that higher sticker valence promotes a sharper conformational collapse as sticker–sticker attraction strengthens. These simulations therefore show that coarse‐grained sticker–spacer models can quantitatively capture the conformational transitions observed experimentally and provide a microscopic basis for understanding how tunable sticker–sticker interactions regulate CGT in associative polymer systems.

### 2.3 Sticker‐Valence‐ and Salt‐Dependent Interfacial Cohesion

**FIGURE 4:**
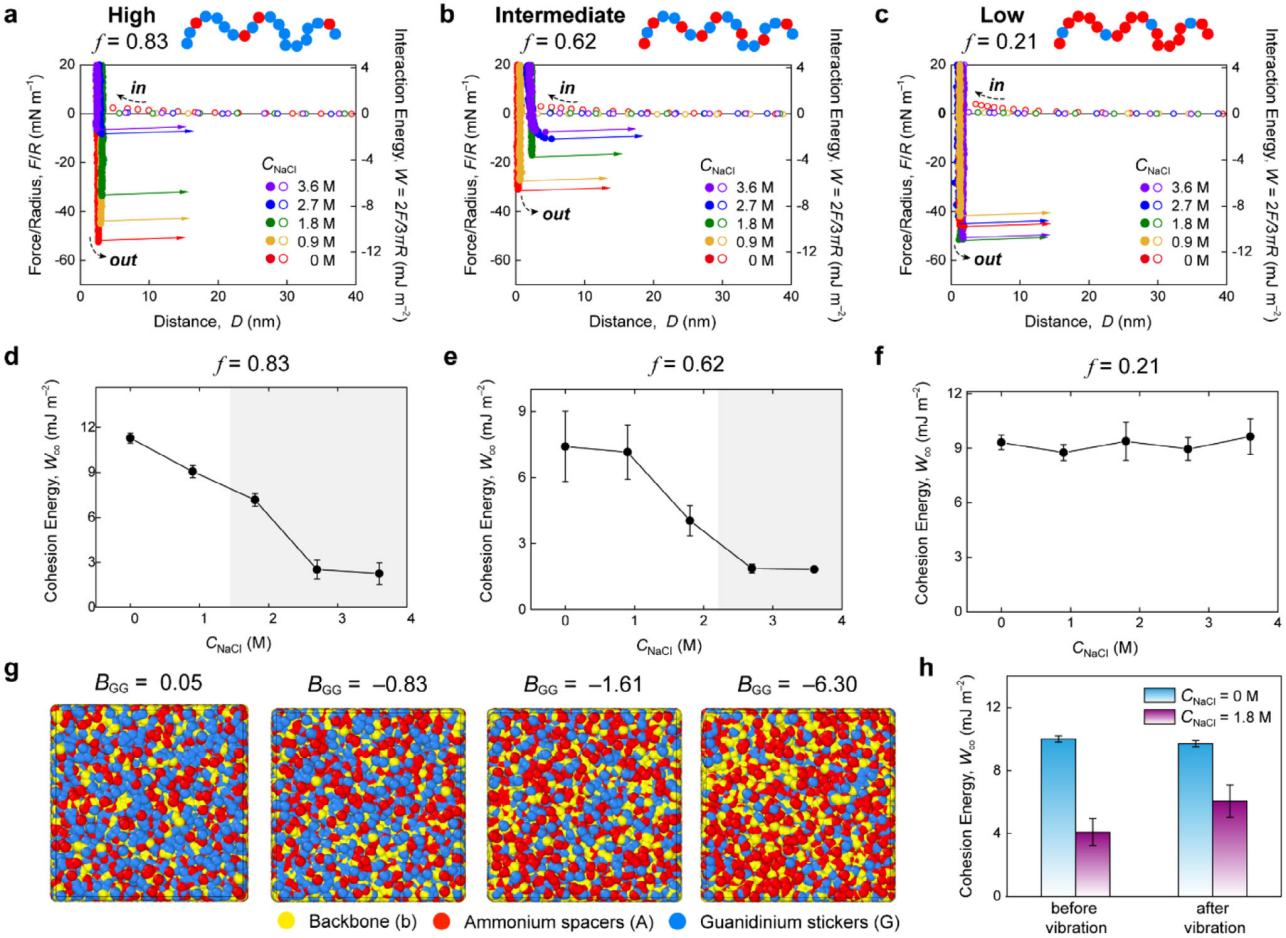
Force–distance profiles during approach and separation of symmetric poly(G‐*ran*‐A) surfaces with *f* = (a) 0.83, (b) 0.62, and (c) 0.21 at *C*_NaCl_ = 0 (red), 0.9 (orange), 1.8 (green), 2.7 (blue), and 3.6 m (purple). Filled and open symbols denote the approach and separation process, respectively. Cohesion energy (*W*_co_) of symmetric poly(G‐*ran*‐A) surfaces with *f* = (d) 0.83, (e) 0.62, and (f) 0.21 as a function of *C*_NaCl_. Error bars and shaded regions denote standard deviation (*n* = 5) and the biphasic phase‐separated regimes in dilute solutions, respectively. (g) Top‐view snapshots of the configuration of sticker−spacer polymer films (*N* = 128, *f* = 0.50) at *B*_GG_ = 0.05, −0.83, −1.61, and −6.30 after equilibrium. (h) Changes in the *W*_co_ after applying high‐frequency vibration to contacting symmetric poly(G‐*ran*‐A) surfaces (*f* = 0.62) at *C*_NaCl_ = 0 (blue) and 1.8 m (purple). Error bars denote standard deviation (*n* = 3).

In dilute solutions, the LLPS and CGT behavior of poly(G‐*ran*‐A) is governed primarily by intermolecular sticker–sticker associations. To directly quantify the corresponding cohesive interaction energy, we employed a miniature surface forces apparatus (µSFA) to measure forces between symmetric poly(G‐*ran*‐A) surfaces with varying sticker fractions (*f* = 0.00, 0.14, 0.21, 0.39, 0.44, 0.62, 0.83, 0.92, and 1.00) over a range of *C*_NaCl_ values (Figure 4a–c and Figure S17) [84]. Even under salt‐free conditions (*C*_NaCl_ = 0), measurable cohesion energies (*W*_co_) were observed for all *f*, including the sticker‐free polymer (*f* = 0.00) (Figure 4d–f and Figure S18). These observations suggest that interfacial attraction can arise even in the absence of added salt through cooperative hydrogen‐bonding interactions among the A and G groups and the polyether backbone, together with possible cation–*π* and *π*–*π* interactions that become accessible upon surface contact and compression (Figure S19) [85, 86].

The salt dependence of *W*_co_ showed distinct trends depending on sticker fraction. We therefore classified the polymers into three regimes: high (*f* = 1.00, 0.92, and 0.83), intermediate (*f* = 0.62, 0.44, and 0.39), and low (*f* = 0.21, 0.14, and 0.00) sticker fractions. At high sticker fractions, *W*_co_ decreased continuously with increasing salt concentration. For example, at *f* = 0.83, *W*_co_ decreased from 11.30 ± 0.33 mJ m^−2^ at *C*_NaCl_ = 0 m to 2.26 ± 0.72 mJ m^−2^ at *C*_NaCl_ = 3.6 m (Figure 4d), with similar trends observed for *f* = 0.92 and 1.00 (Figure S18a,b). At intermediate sticker fraction, *W*_co_ exhibited a plateau at low salt concentration and then decreased as *C*_NaCl_ approached the LLPS threshold. For example, at *f* = 0.62, *W*_co_ remained nearly constant below 1 m NaCl, but decreased to 1.83 ± 0.11 mJ m^−2^ at 3.6 m (Figure 4e). Similar trends were observed for *f* = 0.44 and 0.39 (Figure S18c,d). By contrast, at low sticker fraction (*f* ≤ 0.21), *W*_co_ remained nearly invariant over the entire salt concentration range (Figure 4f and Figure S17e,f).

Counterintuitively, the decrease in *W*_co_ became pronounced at salt concentrations near or above *C*_cr_, particularly for intermediate‐to‐high sticker fractions. We attribute this behavior to preferential intra‐surface sticker–sticker associations, which compete with inter‐surface cohesion upon contact, consistent with our previous work [63], and related observations in mussel‐derived peptide systems [87]. Because positively charged poly(G‐*ran*‐A) chains adsorb densely onto negatively charged mica through electrostatic interactions, the effective polymer concentration at the surface is much higher than in bulk dilute solution. Under these conditions, increasing salt concentration enhances the association strength *λ*_att_, and together with the elevated local monomer concentration increases the association probability *p*. As a result, dense intra‐surface sticker–sticker crosslinking is promoted, forming gel‐like coacervate layers on each surface while reducing the number of free stickers available for inter‐surface cohesion. This provides a direct explanation for the observed reduction in *W*_co_ at high ionic strength [87].

To further probe this mechanism, we performed coarse‐grained MC‐GL simulations of sticker–spacer polymer films confined to an attractive bottom surface (Figure S15). The simulated polymers consisted of G sticker beads and A spacer beads attached to backbone beads, with *f* = 0.50 and *N* = 128, consistent with the CGT simulations described above (Section 4.2). Under these conditions, we quantified the number of sticker beads exposed at the upper free surface as a function of *B*_GG_ (Figure S16a–e). As the sticker–sticker interaction strength increased, that is, with increasingly negative *B*_GG_, the number of surface‐accessible sticker beads progressively decreased (Figure S16f). This depletion was also evident in the top‐view snapshots (Figure 4g), where sticker beads become less prevalent at the interface. These results directly support the µSFA measurements: stronger sticker–sticker association promotes intra‐surface binding and thereby reduces the number of free stickers available for inter‐surface cohesion.

The kinetic origin of this behavior is further supported by the bond lifetime of sticker–sticker associations. The lifetime (*τ*) scales exponentially with interaction energy as *τ* ∼ *τ*_0_ exp (*E*_a_/*k*_B_ *T*), analogous to *λ*_att_, where *τ*_0_ is an attempt time for thermal motion [88, 89]. At high ionic strength, strongly associated sticker–sticker bonds therefore become effectively irreversible on the experimental timescale. In this regime, thermal fluctuations alone are insufficient to break the physical crosslinks between stickers, and the surface‐condensed layer becomes kinetically trapped. To test whether these kinetically trapped intra‐surface associations could be disrupted mechanically, we applied high‐frequency surface vibration (10 kHz, 1 nm amplitude) to contacting poly(G‐*ran*‐A) surfaces with *f* = 0.62 using a piezoelectric tube. Measurements were performed under both salt‐free and 1.8 M NaCl conditions (Figure S20). At 1.8 m NaCl, *W*_co_ increased from 4.09 ± 0.86 to 6.07 ± 1.04 mJ m^−2^ after vibration (Figure 4h), indicating that mechanical stimulation transiently enhanced inter‐surface cohesion by disrupting intra‐surface associations and exposing additional stickers for interfacial binding. In contrast, *W*_co_ remained essentially unchanged in salt‐free solution. After separating the surfaces and allowing them to equilibrate for approximately 1 h, *W*_co_ returned to its pre‐vibration value in 1.8 m NaCl, indicating reformation of intra‐surface *π*–mediated associations over time. Moreover, the results for the high sticker fraction (*f* = 0.92) and low sticker fraction (*f* = 0.14) further support the presence of intra‐surface associations driven by the sticker–sticker bonding (Figures S21 and S22). For the high‐*f* poly(G‐*ran*‐A), *W*_co_ remained nearly unchanged in salt‐free solution, whereas it increased significantly from 5.29 ± 0.53 to 7.32 ± 0.55 mJ m^−2^ after vibration in 1.8 m NaCl. In contrast, low‐*f* poly(G‐*ran*‐A) exhibited no measurable changes in *W*_co_ under any tested conditions. These results show that once intra‐surface associations are established, mere surface contact is insufficient to induce chain rearrangement, whereas external mechanical perturbation can transiently overcome this kinetic barrier and promote reversible interfacial cohesion.

Collectively, these results show that salt‐enhanced guanidinium association drives surface‐localized gelation in poly(G‐*ran*‐A), leading to reduced inter‐surface cohesion despite stronger sticker–sticker attraction at the molecular level. This association‐induced curing behavior provides a mechanistic basis for understanding how ionic environment and sticker valence control interfacial mechanics in associative polymer films. More broadly, guanidinium‐containing polymers with tunable sticker valence and association strength emerge as promising building blocks for bio‐inspired functional materials, including wet adhesion, interfacial sealants, and underwater coatings.

## 3 Conclusion

We established a quantitative molecular framework that links sticker valence, chain conformation, interfacial association, and liquid‐liquid phase separation (LLPS) in peptide‐inspired sticker–spacer heteropolymers bearing Arg‐like guanidinium motifs. By systematically varying the guanidinium fraction in poly(G‐*ran*‐A), we showed that higher sticker valence lowers the critical salt concentration for LLPS and promotes reversible associative phase separation. Prior to macroscopic phase separation, increasing ionic strength induces a continuous coil‐to‐globule transition (CGT) through intrachain sticker association, even while the spacer segments and polyether backbone remain under overall good‐solvent conditions. Consistently, the theta salt concentration decreased with increasing sticker valence, showing that stronger and more abundant sticker interactions effectively lower the apparent solvent quality and shift the conformational transition to lower salt concentration. At interfaces, µSFA measurements, coarse‐grained simulations, and vibration experiments further revealed that stronger guanidinium‐mediated association promotes preferential intra‐surface crosslinking, thereby reducing the number of free stickers available for inter‐surface cohesion. These results show that sticker valence governs not only bulk phase behavior but also interfacial mechanics under high ionic strength. Overall, this work provides molecular insight into Arg‐driven associative phase behavior and establishes poly(G‐*ran*‐A) as a tunable bio‐inspired model system for investigating programmable coacervates and responsive soft materials.

## 4 Materials and Methods

### 4.1 Experimental Section

#### 4.1.1 Polymer Synthesis

Random copolymers composed of guanidinium stickers and ammonium spacers, denoted poly(G‐*ran*‐A), with varying sticker fractions (*f*) were synthesized via living anionic ring‐opening polymerization, followed by post‐polymerization modifications, as previously reported (Figure S1) [63, 64]. Under an argon atmosphere, dried benzyl alcohol (TCI), and tetrahydrofuran (THF, J. T. Baker) were added to a flame‐dried reactor, and potassium naphthalenide was introduced to initiate polymerization. Purified allyl glycidyl ether (AGE, TCI) was then introduced and polymerized at 40°C for 20 h with vigorous stirring. The reaction was quenched with anhydrous methanol and poly(allyl glycidyl ether) (PAGE) was isolated by precipitation in *n*‐hexane. The dispersity (*Ð* = 1.09) and number‐average degree of polymerization (*N*_n_ = 130) of PAGE were determined by size exclusion chromatography (SEC, JASCO, Japan) and proton nuclear magnetic resonance spectroscopy (^1^H NMR, Unity‐Inova 500, Varian, USA), respectively (Figure S2 and S3). Ammonium (A) groups were introduced to the double bond in PAGE via a thiol‐ene click reaction. PAGE, 2‐aminoethanethiol hydrochloride (thiol agent, TCI, 4 equiv. per monomer), and 2,2‐dimethoxy‐2‐phenylacetophenone (DMPA, photo initiator, Sigma–Aldrich, 0.05 equiv. per monomer) were dissolved in a mixture of water/methanol. The solution was purged with argon and exposed to UV light (365 nm) for at least 12 h under stirring. Residual reactants were removed by dialysis using a regenerated cellulose tubular membrane (molecular weight cut‐off: 6–8 kDa) against deionized water for 5 days, and the resulting ammonium‐functionalized PAGE, denoted poly(A), was recovered by lyophilization. Subsequently, the A groups were converted into guanidinium (G) groups via a guanylation reaction using 1H‐Pyrazole‐1‐carboxamidine hydrochloride (TCI, 5 equiv. per monomer) in a basic aqueous solution (pH = 12), followed by dialysis and lyophilization. The guanidinium mole fraction (*f*) was characterized as a function of reaction time by analyzing the peak area ratio of A and G groups in the ^1^H NMR spectra (Figures S4 and S5).

#### 4.1.2 Optical Measurements

Liquid‐liquid phase separation (LLPS) droplets were observed by optical microscope (BX51, Olympus, Japan). Poly(G‐*ran*‐A) solutions with a polymer concentration of 0.1 wt.% at *C*_NaCl_ = 0 and 2.0 m were vortexed for 10 s and then mounted between a glass slide and a coverslip. For transmittance measurements, 0.1 wt.% aqueous poly(G‐*ran*‐A) solutions were loaded into disposable cuvettes. Transmittance at a wavelength of 600 nm was measured using a UV‐vis spectrophotometer (8453, Agilent Technologies, USA). The temperature was maintained at 23°C using a Peltier temperature controller (TC1, Quantum Northwest, USA). Poly(ethylene glycol) methyl ether (PEG, *M*_n_ = 10,000 g mol^–^^1^, Sigma–Aldrich) and 1,6‐hexanediol (Sigma–Aldrich), and guanidinium hydrochloride (GdmCl, Sigma–Aldrich) were used as a molecular crowder, a hydrophobic interaction disruptor, and a sticker interaction inhibitor, respectively.

#### 4.1.3 Small‐Angle Neutron Scattering (SANS)

Small‐angle neutron scattering (SANS) measurements were performed using the 40 m‐SANS instrument at the High‐flux Advanced Neutron Application ReactOr (HANARO), Korea Atomic Energy Research Institute (KAERI) [90]. The instrument was operated with a neutron wavelength of *λ* = 6 Å, a wavelength spread of ∆*λ*/*λ* = 0.12, and sample‐to‐detector distances of 7 and 1.16 m, covering a scattering wave vector (*q*) range of 0.01 Å^−1^< *q* < 0.48 Å^−1^, where *q* = (4*π*/*λ*) sin(*θ*/2) and *θ* is the scattering angle. The temperature was maintained at 23°C using a circulating water bath. Aqueous solutions of 0.1 wt.% poly(G‐*ran*‐A) and poly(ethylene glycol) methyl ether (PEG, *M*_n_ = 5,000 g mol^−1^, Sigma–Aldrich), prepared in D_2_O, were loaded into quartz banjo cells with a 2 mm path length and exposed to the neutron beam. The resulting two‐dimensional isotropic scattering data were azimuthally averaged, and the scattering intensity was reduced to an absolute scale using the direct beam flux method. The coherent scattering intensity was obtained by subtracting the incoherent solvent scattering.

The SANS profiles were fitted to extract the radius of gyration (*R*_g_) and the effective Flory exponent (*ν*_eff_) using the form factor *P*(*q*), given by [82]:

(2) $$P\left(q\right)=\frac{1}{\nu_{\mathrm{eff}}U^{1/2\nu_{\mathrm{eff}}}}\gamma \left(\frac{1}{2\nu_{\mathrm{eff}}},\;U\right)-\frac{1}{\nu_{\mathrm{eff}}U^{1/\nu_{\mathrm{eff}}}}\gamma \left(\frac{1}{\nu_{\mathrm{eff}}},\;U\right)$$

where *γ*(*x*, *U*) is the incomplete gamma function, defined as:

(3) $$\gamma \left(x,\;U\right)=\underset{0}{\int^{U}}dt\exp\left(-t\right)t^{x-1}$$

The variable *U* is expressed in terms of the scattering vector *q* as:

(4) $$U=\frac{q^{2}{R_{g}}^{2}\left(2\nu_{\mathrm{eff}}+1\right)\left(2\nu_{\mathrm{eff}}+2\right)}{6}$$

In the limit of *ν*_eff_ = 0.5, *P*(*q*) reduces to the Debye form factor *D*(*q*).

(5) $$D\left(q\right)=\frac{2}{q^{4}{R_{g}}^{4}}\left[\exp\left(-q^{2}{R_{g}}^{2}\right)-1+q^{2}{R_{g}}^{2}\;\right]$$

#### 4.1.4 Static Light Scattering (SLS)

Static light scattering (SLS) measurements were performed using a BI‐200SM goniometer (Brookhaven Instruments, USA) equipped with a laser (*λ* = 639 nm). Poly(G‐*ran*‐A) solutions (*f* = 0.35) with concentrations ranging between 5 and 30 mg mL^−1^ was filtered through 0.45 µm poly(vinylidene fluoride) (PVDF) filters (HYUNDAI MICRO) and loaded into glass tubes with a diameter of 10 mm. These sample tubes were placed in a reservoir filled with dust‐free decalin, and the temperature was maintained at 23°C using a circulating bath.

Scattering angles between 45° and 140° were measured in 5° increment, corresponding to 0.0010 Å^−1^< *q* < 0.0052 Å^−1^. Within this *q* range, the scattering intensity was essentially independent of *q*, indicating that *qR*_g_ ≪ 1 under the present conditions (Figure S11). In this Rayleigh scattering regime, the weight‐average molecular weight (*M*_w_) and the effective osmotic second virial coefficients (*B*_eff_) were determined using

(6) $$\frac{\mathrm{Kc}}{R_{\theta }}=\frac{1}{M_{w}}+2B_{\mathrm{eff}}c$$

where *c* is the mass concentration of the solution and *K* is the optical constant defined as *K* = 4*π* ^2^ *n*_0_^2^(d*n*/d*c*)^2^/*N*_A_ *λ* ^4^. Here, *n*_0_, d*n*/d*c* and *N*_A_ denote the refractive index of solvent, the refractive index increment of the solution, and Avogadro`s number, respectively. *R*_θ_ is the normalized excess scattering intensity per unit volume, that is, the Rayleigh ratio, calibrated using anhydrous toluene. The refractive indices of poly(G‐*ran*‐A) solutions and solvents were measured as a function of *c* using a digital refractometer (Abbemat 300, Anton Paar, Austria) at 23°C (Figure S12), from which dn/dc values were obtained (Table S1). The measured *K* *c*/*R*_θ_ values were averaged over all *q* values and plotted as a function of *c* to determine *B*_eff_ as a function of *C*_NaCl_ (Figure S13).

#### 4.1.5 Miniature Surface Forces Apparatus (µSFA)

The interaction energies between symmetric poly(G‐*ran*‐A) surfaces were investigated using a miniature surface forces apparatus (µSFA, SurForce LLC, USA) [84, 91]. SFA technique has long been used to measure interaction forces between synthetic and biopolymers [85, 92, 93, 94, 95]. Prior to the experiments, the polymers were dissolved in the deionized (DI) water at a polymer concentration of 1 µg mL^−1^ and filtered through a 0.2 µm poly(tetrafluoroethylene) (PTFE) filter (ADVANTEC) to remove impurities. Freshly cleaved muscovite mica (Grade #1, S&J Trading, USA) was used as the substrate, and a 50 nm silver layer was deposited on the mica. The back‐silvered mica was then gently glued onto cylindrical disks with a radius of curvature of 2 cm using a UV‐cured optical adhesive (NOA 81, Norland Products Inc., USA), followed by curing for ca. 1 h [91]. The polymer solution was drop‐cast onto the mica surface for 10 min, rinsed with DI water to remove unbound polymer, and dried under a stream of N_2_. The prepared polymer‐coated surfaces were mounted in the µSFA chamber in a crossed‐cylinder geometry, and either DI water or NaCl solution (∼50 µL) was injected between the opposing surfaces. Measurements were performed at *C*_NaCl_ = 0, 0.9, 1.8, 2.7, 3.6 m. To minimize evaporation during the measurements, the chamber was sealed with 2 mL of DI water, and the surfaces were equilibrated for ∼1 h prior to measurements.

Force measurements were performed at a constant displacement rate of ∼5 nm s^−1^ by approaching and retracting the two opposing surfaces. The surfaces were compressed to hard contact and retracted after a contact time of 5 s. The interaction force (*F*) was determined from the deflection of a double‐cantilever spring (*k* = 1961.3 N m^−1^) supporting the lower disk. The mica‐to‐mica distance was measured from the fringes of equal chromatic order (FECO) using multiple‐beam interferometry (MBI) with a white light source [96]. The minimum *F*/*R* value obtained during retraction, corresponding to the cohesion force between the surfaces, was converted to the cohesion energy per unit area using the Johnson‐Kendall‐Roberts model according to *W*_co_ = 2*F*/3*π* *R* [91, 97]. All measurements were conducted at room temperature (*T* = 22.5°C) and repeated five times.

To apply high‐frequency vibration to the contacting polymer surfaces, the piezoelectric tube supporting the upper disk was used for fine displacement control. The voltage‐to‐distance of the piezoelectric crystal was calibrated to be approximately 1 nm V^−1^ at separation greater than 400 nm, where surface interactions were negligible [98]. When the surfaces were compressed up to ∼10 mN m^−1^, the piezoelectric tube vibrated in a 1 nm amplitude triangle function at a frequency of 10 kHz for 5 min. The surfaces were then retracted at a constant speed of ∼5 nm s^–^^1^, and the detached surfaces were equilibrated at a separation greater than 400 nm. To confirm the reproducibility, a cycle consisting of (i) force measurement without vibration, (ii) force measurement with vibration, and (iii) 1 h equilibration was repeated three times.

### 4.2 Computational Methods

#### 4.2.1 Theoretically Informed Coarse‐Grained (TICG) Simulations

The sticker−spacer model consists of backbone polymer chains composed of coarse‐grained beads interconnected by Finite Extensible Nonlinear Elastic (FENE) bonds. Each backbone bead carries a side unit, which is randomly assigned as either stickers, capable of strong associative interactions, or a non‐associative spacer. Each side unit is represented by a coarse‐grained bead connected to the corresponding backbone bead by a harmonic spring. The bonding potential of side units (*H*_b, sc_) at a given temperature, *T*, is given by:

(7) $$H_{b,\mathrm{sc}}=\frac{3k_{B}T}{2}\frac{N-1}{{R_{e,\mathrm{ideal}}}^{2}}\sum_{k=1}^{n}\sum_{j=1}^{N_{\mathrm{bb}}}b_{k}^{2}\left(i\right)$$

where *b*_k_(*i*) is the bond vector between the *i* ^th^ backbone bead and its attached side bead in the *k* ^th^ chain among *n* sticker−spacer polymer chains. *N*_bb_ represents the total number of backbone beads, *R*_e, ideal_ is the ideal root ‐ mean‐squared end‐to‐end distance of a linear chain with *N* beads, and *k*_B_ is the Boltzmann constant. The reference chain length *N* was set to be 32.

The FENE potential was used to model bonding interactions between the backbone beads, allowing us to capture the rigidity of the backbone chain. The average bond length between adjacent backbone beads, *b*_bb_ = 0.17961*R*_e, ideal_, was chosen to be identical to the average bond length of the side chains, *b*_sc_. The FENE bonding potential within backbones (*H*_b, bb_) is given by:

(8) $$H_{b,\mathrm{bb}}=-\sum_{k=1}^{n}\sum_{i=1}^{N_{\mathrm{bb}}}\frac{1}{2}k_{s}r_{0}^{2}\ln\left(1-\left.{\left(\frac{r_{k}\left(i\right)-b_{\mathrm{bb}}}{r}\right)}^{2}\right)\right.\;$$

where *r*_k_(*i*) is the distance between the *i* ^th^ and (i + 1)^th^ backbone beads in the *k* ^th^ polymer chain. The spring constant was set to *k*_s_ = 1000*k*_B_ *T*/*R*_e, ideal_^2^, and *r*_0_ is the maximum allowable distance between backbone beads, chosen as 1.5 times the equilibrium backbone bond length *b*_bb_.

The non‐bonded Hamiltonian (*H*_nb_) was described as a functional of density fields up to the third order in a virial expansion framework [99]:

(9) $$H_{\mathrm{nb}}=k_{b}\;T\int_{V}\frac{d^{3}r}{{R_{e,\mathrm{ideal}}}^{3}}\left(\sum_{\alpha =1}^{m}\sum_{\beta =1}^{m}\frac{B_{\alpha \beta }\rho_{1\alpha }\rho_{2\beta }}{2}+\sum_{\alpha =1}^{m}\sum_{\beta =1}^{m}\sum_{\gamma =1}^{m}\frac{\omega_{\alpha \beta \gamma }\rho_{1\alpha }\rho_{3\beta }\rho_{3\gamma }}{3}\right)$$

where the integration is performed over the entire simulation box volume (*V*), and the summations within the integral are taken over all types of species (*m*) in the system. The terms *ρ*_1α_, *ρ*_2α_, and *ρ*_3α_ denote the number‐weighted first, second, and third local densities of a particular species *α*, calculated using a zeroth‐order Particle‐to‐Mesh (PM0) scheme, where the entire bead weight is allocated to the nearest grid point [100]. *B*_αβ_ and *ω*_αβγ_ are the second‐ and third‐order interaction coefficients, respectively. The cross‐second order virial coefficient was defined as *B*_αβ_ = (*χ*_αβ_ *N*)/*ρ* + (*B*_αα_ + *B*_ββ_)/2 and the cross‐third order virial coefficient *ω*_αβγ_ were taken as the arithmetic average of third order self‐interaction coefficients. To isolate the effect of sticker interactions, the interaction parameters for spacers and backbone beads were chosen to represent good‐solvent conditions, while *B*_GG_ was varied to tune the attractive interaction between sticker beads.

#### 4.2.2 Dilute‐Solution Single‐Chain Simulations

Single‐chain simulations were carried out for sticker fraction *f* = 0.25, 0.50, 0.75, and 1.00, with backbone chain lengths *N*_bb_ = 32, 64, 128, 256, 512, and 1024. The second virial coefficients for spacer–spacer (*B*_AA_) and backbone–backbone (*B*_bb_) interactions were fixed at *B*_AA_ = *B*_bb_ = 0 to isolate the effect of sticker–sticker association, whereas the values of *B*_GG_ used for each sticker fraction are listed in Table S2. Third‐order virial coefficients were set to *ω*_GGG_ = *ω*_AAA_ = *ω*_bbb_ = 0.04 and *χ*_αβ_ *N* = 0 was used for all three species.

Simulation box size was fixed at 10*R*_e, ideal_ with periodic boundary conditions in the *x*, *y*, and *z* directions. Each simulation was run for a total of 2 × 106 Monte Carlo (MC) steps. After the first 106 equilibration steps, 1000 coordinate datasets were collected to calculate the radius of gyration, and the results were averaged over five independent ensembles. To accelerate equilibration, the MC moves included pivot moves for both backbone and side‐chain segments, segmental translations of side chains, and single‐bead displacements. The maximum displacement for single‐bead moves in the side chains and backbone was set to 0.8 times the average bond length, and the pivoting rotation angle was limited to 1.2 radians. The effective Flory exponent (*ν*_eff_) was obtained from the slope of the fitted line in the log‐log plot of the calculated radius of gyration (*R*_g_) as a function of chain length, *N* (Figure S14) [101].

#### 4.2.3 Polymer‐Film Simulations with a Free Surface and Substrate Attraction

To directly examine the mechanistic origin of the salt‐dependent cohesion behavior in the µSFA measurements, we performed polymer‐film simulations incorporating both a free surface and an attractive substrate that mimics the negatively charged mica surface. In the melt system, the interaction parameters were adjusted relative to the single‐chain simulations to account for dense polymer packing. The second‐order virial coefficients for spacer and backbone beads were set to *B*_AA_ = *B*_bb_ = −1.60938, while the third‐order coefficients were set to *ω*_AAA_ = *ω*_bbb_ = 0.00933. *χ*_αβ_ *N* = 0 was used for all three species. The second‐ and third‐order coefficients for the sticker beads (*B*_GG_, *ω*_GGG_) were systematically varied as listed in Table S3 to reproduce the range of sticker–sticker interaction strengths corresponding to different experimental salt concentrations. The simulated polymers had a sticker fraction of *f* = 0.50 and a backbone length *N*_bb_ = 128, and were placed in a periodic box of size 4*R*_e, ideal_ × 4*R*_e, ideal_ × 3*R*_e, ideal_, periodic in the *x* and *y* directions. For initialization, the first bead of each chain was placed at a random position 0 < *z* < 2*R*_e, ideal_, and the remaining beads were sequentially generated in random directions at the equilibrium bond length *b*_bb_ = *b*_sc_ = 0.17961*R*_e, ideal_. This initialization concentrated the polymer density in the lower region of the box, leaving the upper region (2*R*_e, ideal_ ≤ *z* < 3*R*_e, ideal_) at lower initial density so as to facilitate the formation of a free polymer surface during equilibration. The system was evolved using the same MC moves as described in the previous section for a total of 5 × 105 MC steps. Equilibration was monitored by tracking the total energy, chain center‐of‐mass positions, and density profiles along the *z*‐direction, all of which reached steady‐state values within the first 4 × 105 steps. Over the final 1 × 105 equilibrated steps, coordinate snapshots were recorded every 1 × 104 steps, yielding 10 configurations used for subsequent analysis.

To model the electrostatic attraction between polymer beads and the substrate, a short‐range, one‐body potential (*H*_s_) was applied to each bead relative to the bottom surface at *z* = 0:

(10) $$H_{s}\left(r_{i},\;K\right)=-k_{B}T\frac{\lambda_{K}}{Nd_{s}/R_{e,\mathrm{ideal}}}\exp\left(-\frac{{z_{i}}^{2}}{2{d_{s}}^{2}}\right)$$

where *z*_*i*_ is the vertical distance of *i* ^th^ bead of type *K* (*K* ∈ {A, b, G}) from the bottom of the simulation box. The potential energy decays over a short distance *d*_s_ = 0.15*R*_e, ideal_. The parameter *λ_K_* denotes the interaction strength between substrate and bead type *K*, and was set to −1.0 for all bead types. This choice ensured surface adsorption without causing irreversible trapping or artificial surface crystallization. Under these conditions, the spatial distribution of sticker and spacer beads along the *z*‐axis was analyzed by dividing the simulation box into 100 equal bins of size 4*R*_e, ideal_ × 4*R*_e, ideal_ × 0.03*R*_e, ideal_ and counting the number of sticker and spacer beads in each bin across all sampled frames (Figure S15). This analysis enabled quantification of the number of stickers located near the free surface that could potentially participate in interfacial cohesion in the µSFA geometry. As the sticker–sticker interaction strength increased, that is, as *B*_GG_ became more negative (Table S3), the number of stickers available near the free surface decreased systematically, indicating that more stickers became sequestered into intra‐film associations within the condensed layer.

## Author Contributions

**Sangyeop Lee**: methodology, software. **Soo‐Hyung Choi**: supervision, project administration, validation, writing – review and editing, funding acquisition, investigation, conceptualization. **Seung‐Hwan Oh**: data curation, formal analysis, investigation, methodology, validation, visualization, writing – original draft. **Seunghyun Lee**: data curation, investigation, methodology. **Jinhoon Lee**: methodology, data curation, formal analysis, validation, investigation, visualization, writing – original draft. **Dong Woog Lee**: conceptualization, investigation, funding acquisition, validation, writing – review and editing, project administration, supervision. **Raehyun Jung**: data curation, formal analysis, methodology, software, writing – original draft. **Su‐Mi Hur**: conceptualization, funding acquisition, investigation, project administration, supervision, validation, writing – review and editing.

## Conflicts of Interest

The authors declare no conflicts of interest.

## Supporting information




**Supporting File**: advs77086‐sup‐0001‐SuppMat.pdf.

## Data Availability

The data that support the findings of this study are available from the corresponding author upon reasonable request.
