# Supplementary material for: Sticker Valence Governs Chain Collapse and Liquid‐Liquid Phase Separation in Peptide‐Inspired Associative Polymers
Source: Adv Sci (Weinh). 2026 Aug 18;13(62):e77086. doi: 10.1002/advs.77086 (PMC13483142; doi:10.1002/advs.77086)
Supplement: Supplementary file 1 [file ADVS-13-e77086-s001.pdf]

Supporting Information

Sticker Valence Governs Chain Collapse and Liquid-Liquid Phase Separation in Peptide-Inspired Associative Polymers

Seung-Hwan Oh[1,†], Jinhoon Lee[2,†], Seunghyun Lee[2], Raehyun Jung[3], Sangyeop Lee[4], Su-Mi Hur[3,], Dong Woog Lee[2,*], and Soo-Hyung Choi[1,*]*

1. Polymer Synthesis

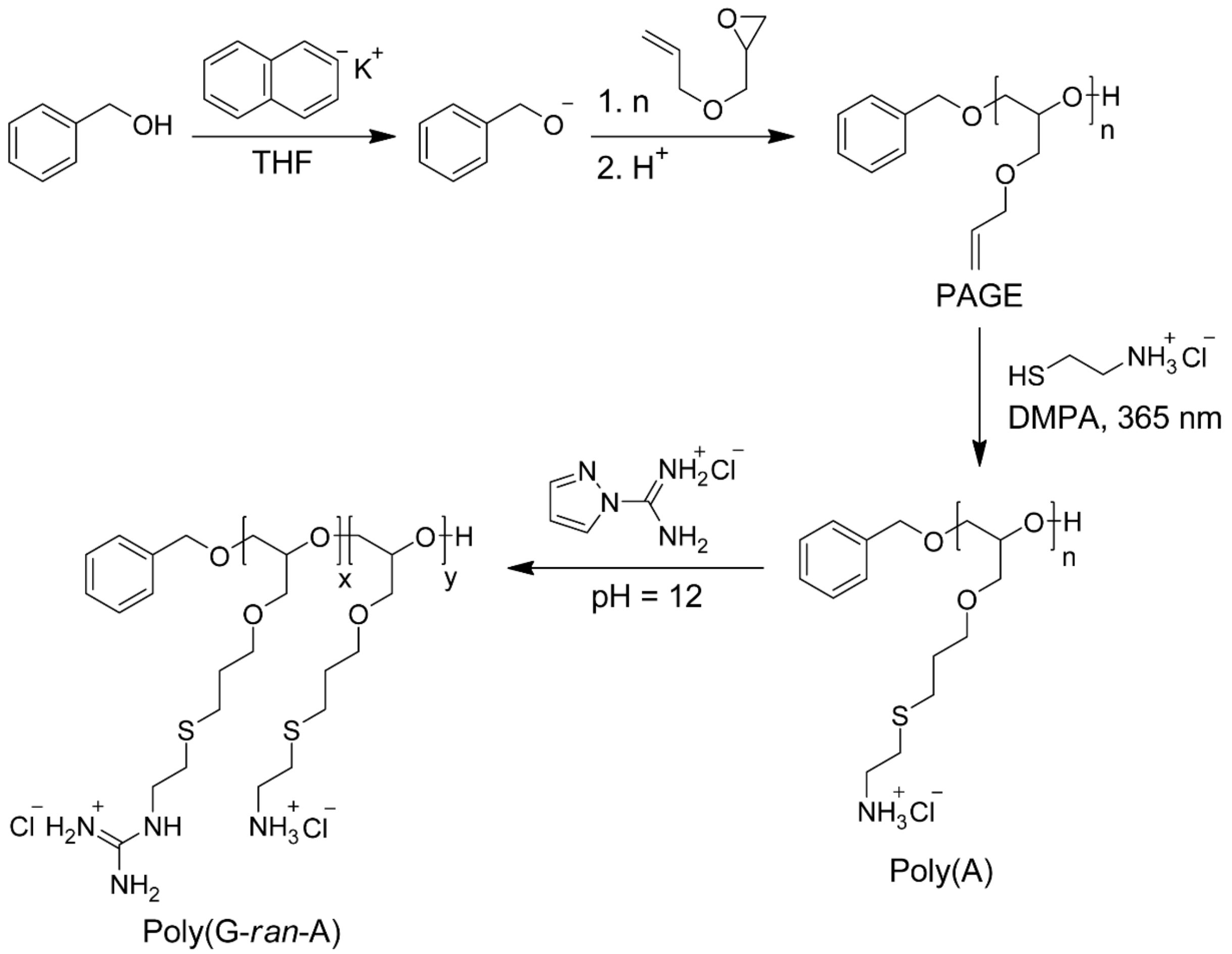

Figure S1. Synthetic route of random copolymers containing guanidinium and ammonium groups, poly(G-*ran*-A), prepared by living anionic ring-opening polymerization of allyl glycidyl ether (AGE) to yield PAGE, followed by post-polymerization modification via thiol-ene click reaction and guanylation.

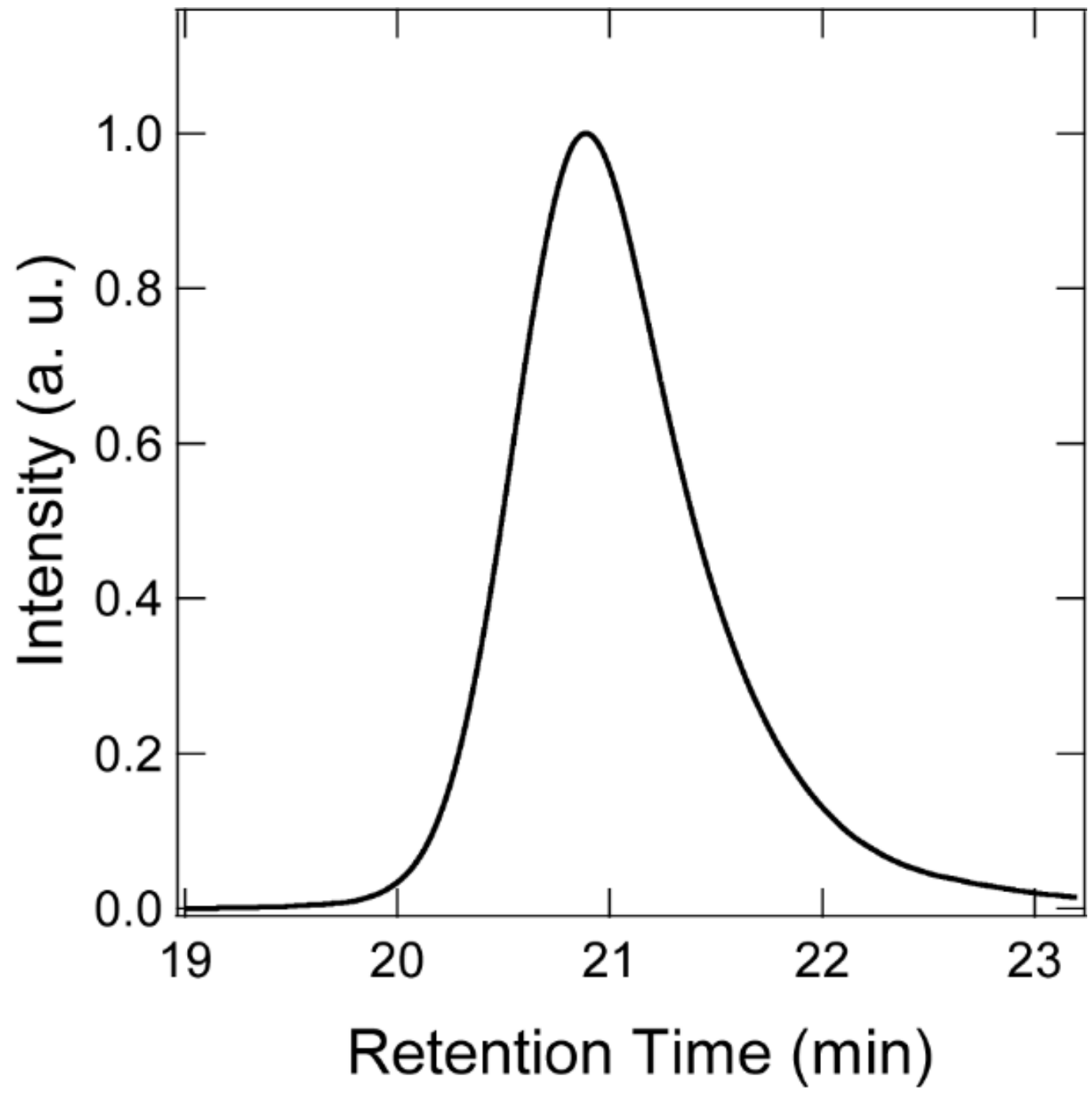

Figure S2. Size exclusion chromatography (SEC) trace of PAGE measured in $CHCl_3$ as an eluent ($Đ$ = 1.09).

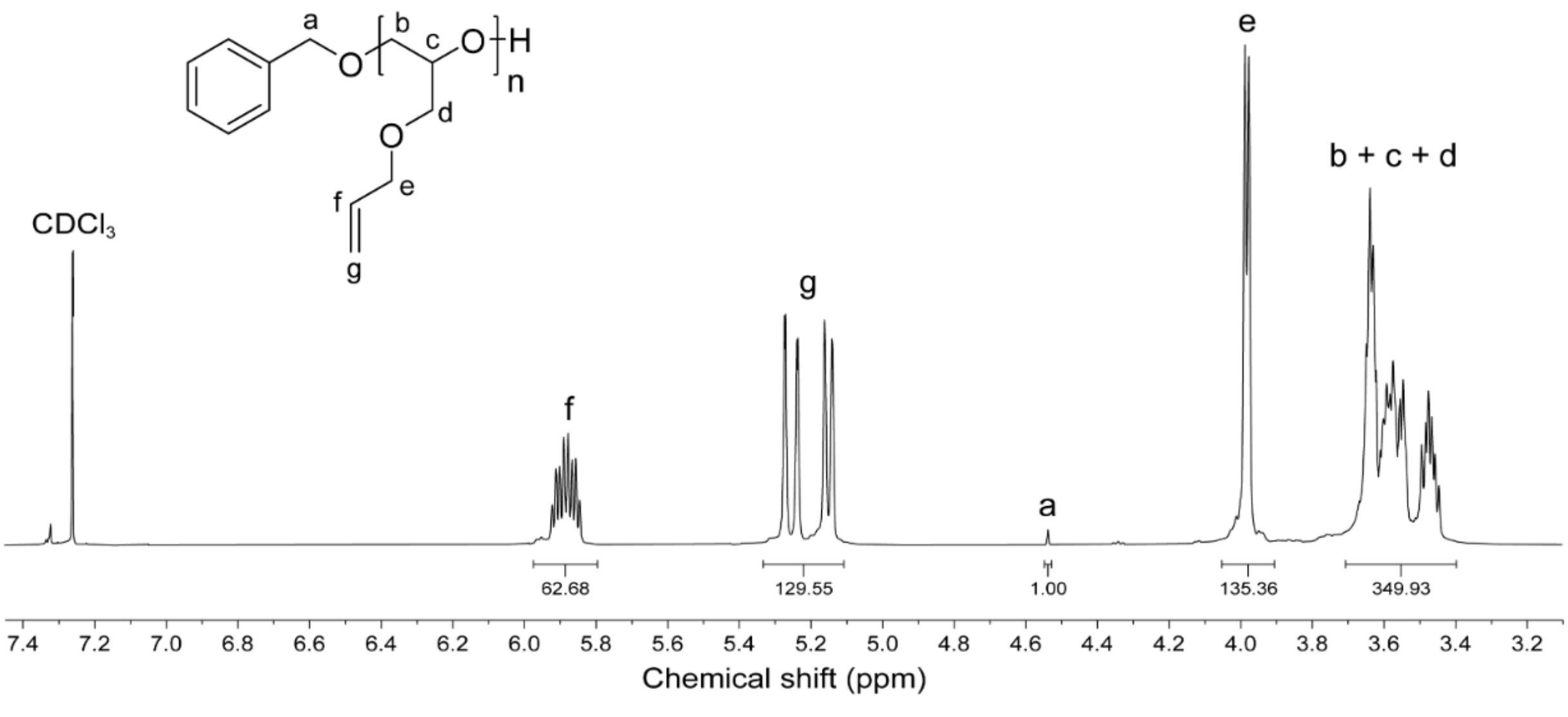

Figure S3. ^{1}H NMR spectrum of PAGE. ^{1}H NMR (500 MHz, $CDCl_3$, room temperature, δ, ppm): 3.40−3.70 (m, 5H, $-OCH_2CHCH_2-$), 4.00 (d, 2H, $-OCH_2CH{=}CH_2$), 4.55 (s, 2H, $-OCH_2C-$), 5.15 (d, 1H, $-OCH_2CH{=}CH_2$), 5.25 (d,1H, $-OCH_2CH{=}CH_2$), 5.90 (m, 1H, $-OCH_2CH{=}CH_2$). The number-average degree of polymerization (N_n = 130) was determined by end-group analysis.

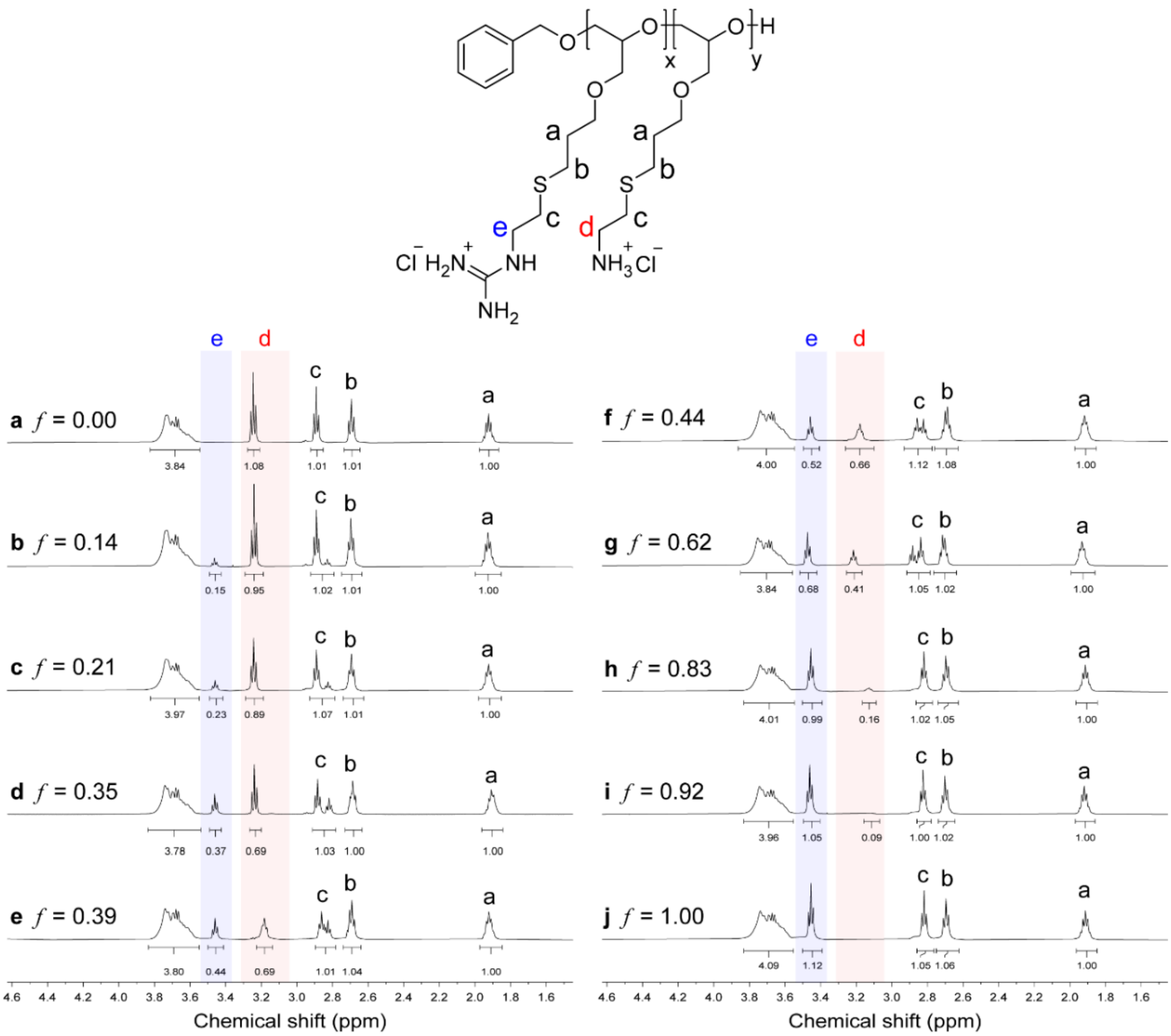

Figure S4. ^{1}H NMR spectra of poly(G-*ran*-A) with sticker fractions (*f*) of (a) 0.00, (b) 0.14, (c) 0.21, (d) 0.35, (e) 0.39, (f) 0.44, (g) 0.62, (h) 0.83, (i) 0.92, and (j) 1.00. ^{1}H NMR (500 MHz, D_2O, room temperature, δ, ppm): 1.90 (t, 2H, $-CH_2CH_2S-$), 2.70 (t, 2H, $-CH_2CH_2S-$), 2.80−2.90 (t, 2H, $-SCH_2CH_2NH_3^+$, $-SCH_2CH_2NH-$), 3.20−3.25 (t, 2H, $-SCH_2CH_2NH_3^+$), 3.45 (t, 2H, $-SCH_2CH_2NH-$), 3.60−3.80 (br, 7H, $-OCH_2CH-$, $-CH_2OCH_2-$). The sticker fraction *f* was determined from the peak areal ratio of the signals assigned to the ammonium (A) and guanidinium (G) groups.

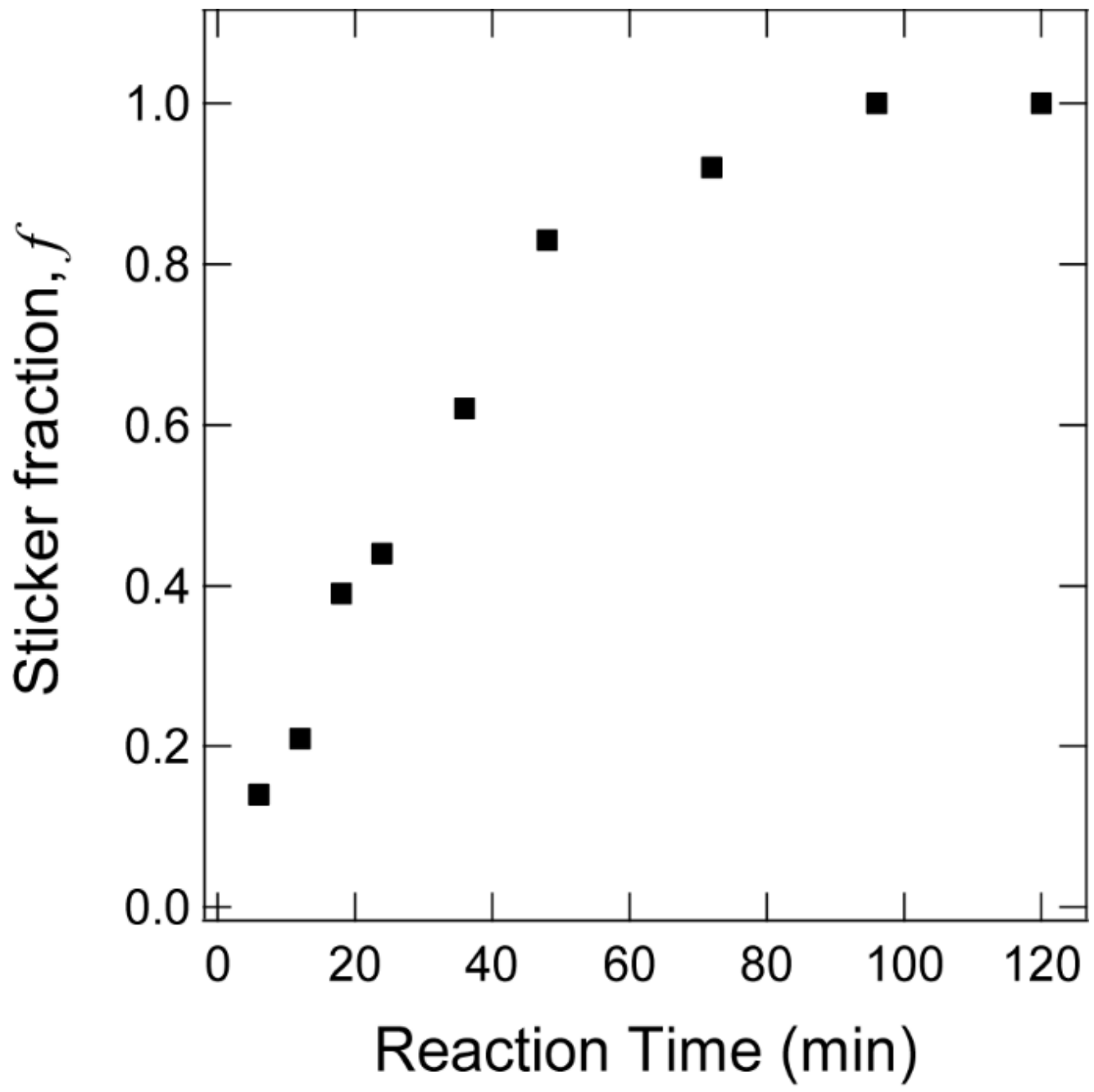

Figure S5. Sticker fraction (*f*) of poly(G-*ran*-A) as a function of guanylation time.

2. Transmittance Measurements

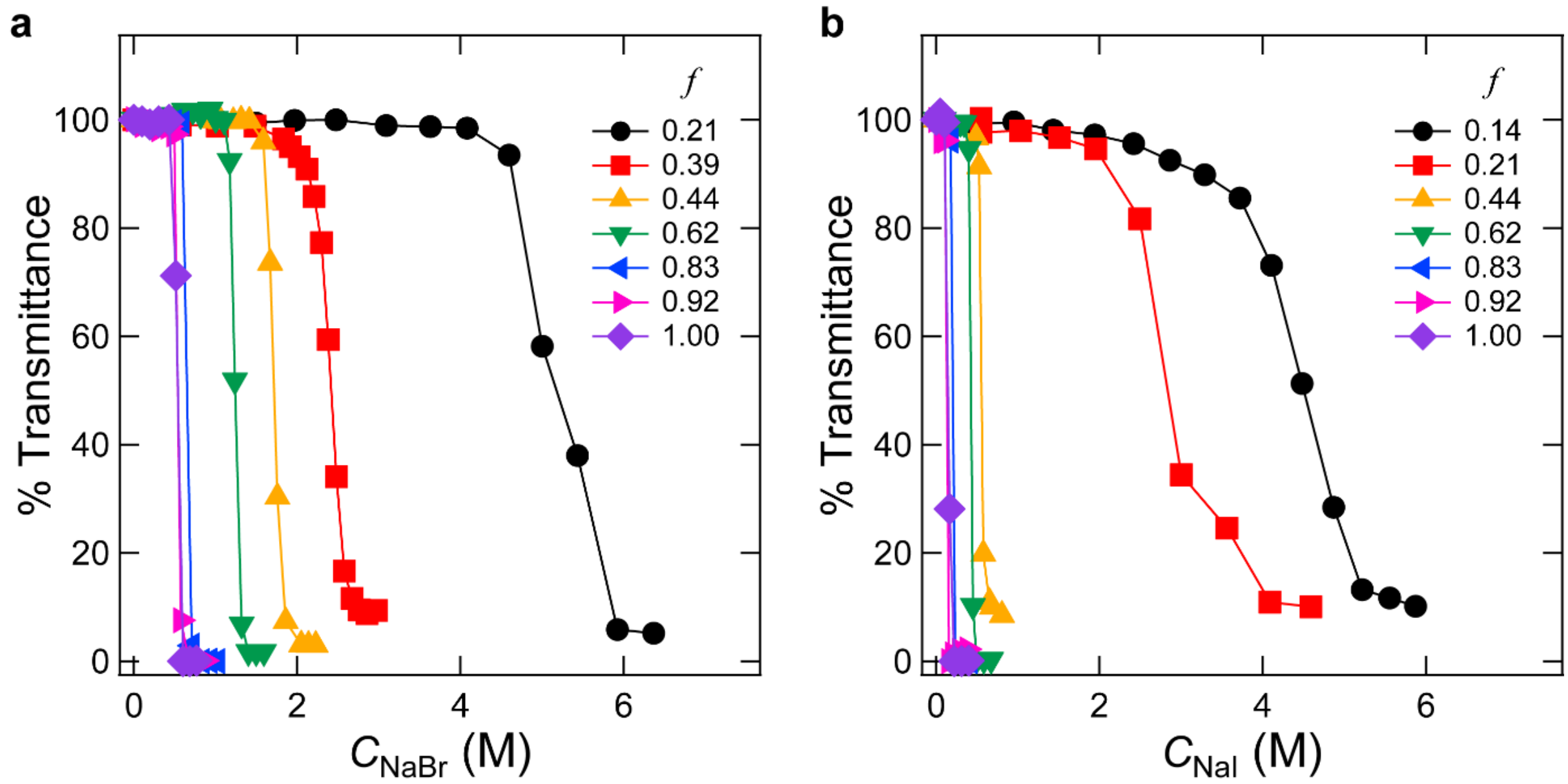

Figure S6. Optical transmittance of 0.1 wt% poly(G-*ran*-A) solutions with varying sticker fraction (f) as a function of (a) NaBr concentration and (b) NaI concentration at 23 ºC. The salt concentrations of NaBr and NaI are denoted as C_{NaBr} and C_{NaI}, respectively.

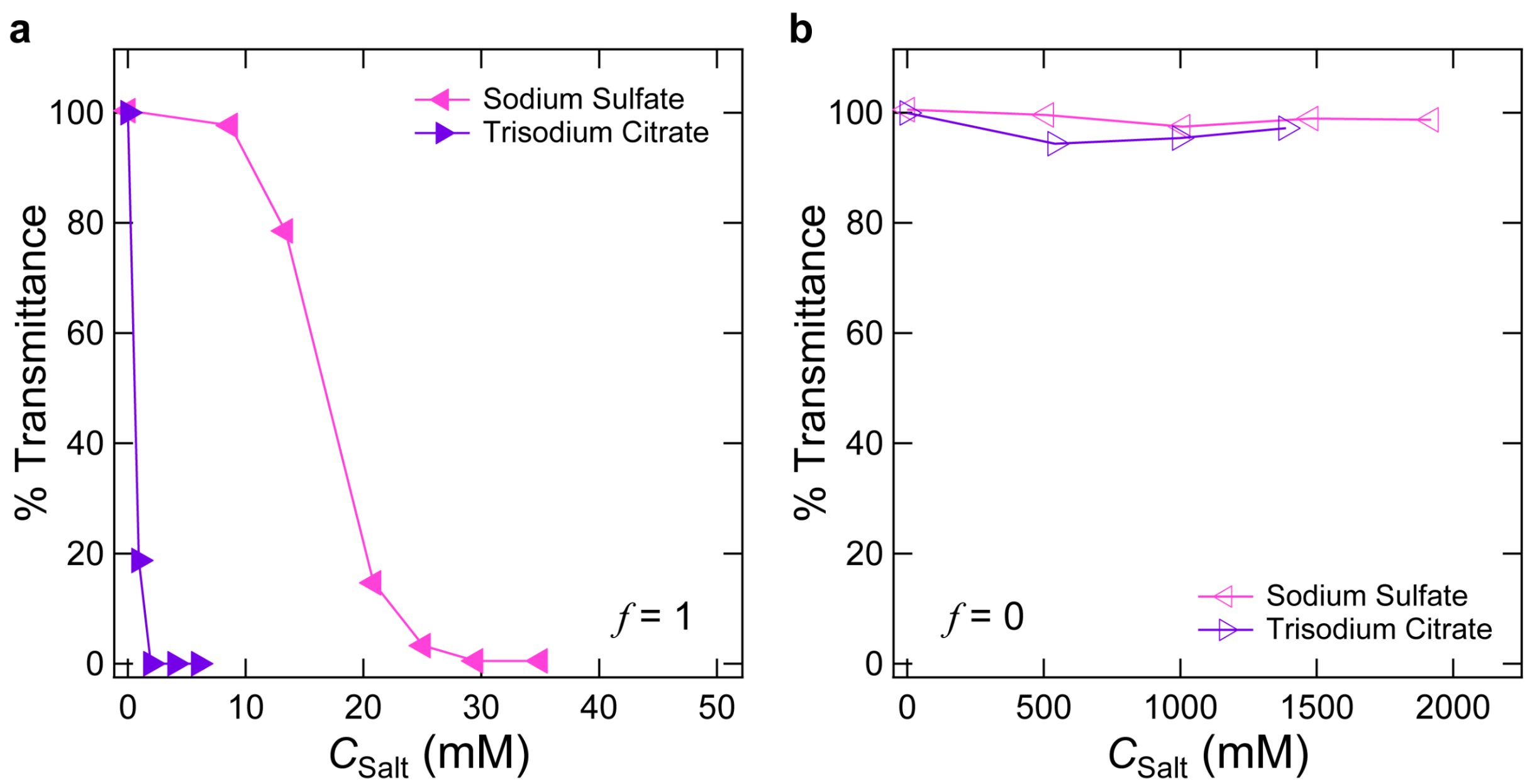

Figure S7. Optical transmittance of 0.1 wt% poly(G-*ran*-A) solutions with (a) $f = 1$ and (b) $f = 0$ as a function of added salt concentration (C_{salt}) for sodium sulfate (magenta) and trisodium citrate (purple) at 23 ºC.

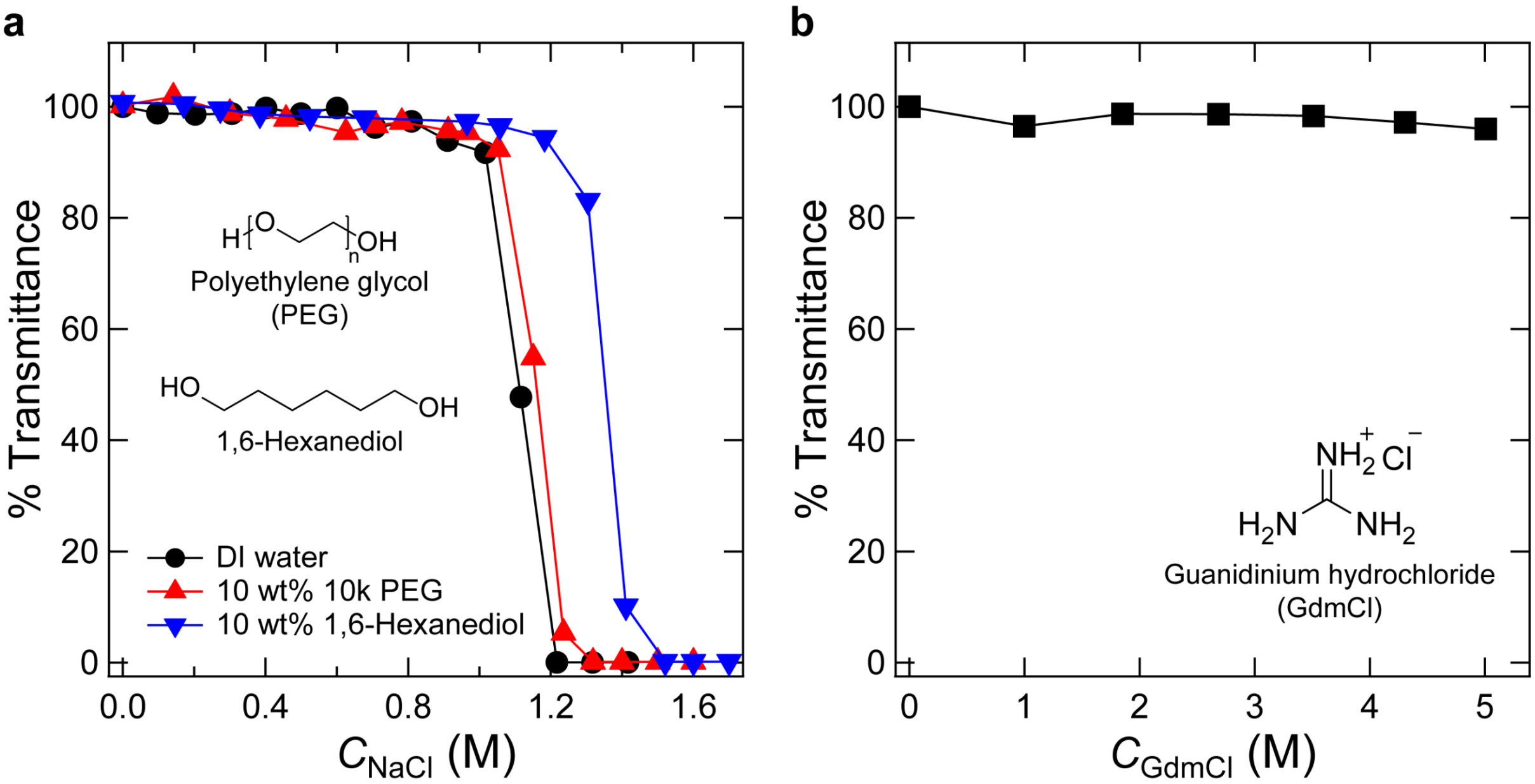

Figure S8. (a) Optical transmittance of 0.1 wt% poly(G-*ran*-A) solutions with f = 1.00 in deionized (DI) water (black), 10 wt% poly(ethylene glycol) (M_n = 10,000 g mol^{-1}, 10k PEG, red), and 10 wt% 1,6-hexanediol aqueous solutions as a function of NaCl concentration (C_{NaCl}) at 23 ºC. (b) Optical transmittance of 0.1 wt% poly(G-*ran*-A) solutions with f = 1.00 as a function of guanidinium hydrochloride concentration (C_{GdmCl}) at 23 ºC.

3. Small-Angle Neutron Scattering (SANS)

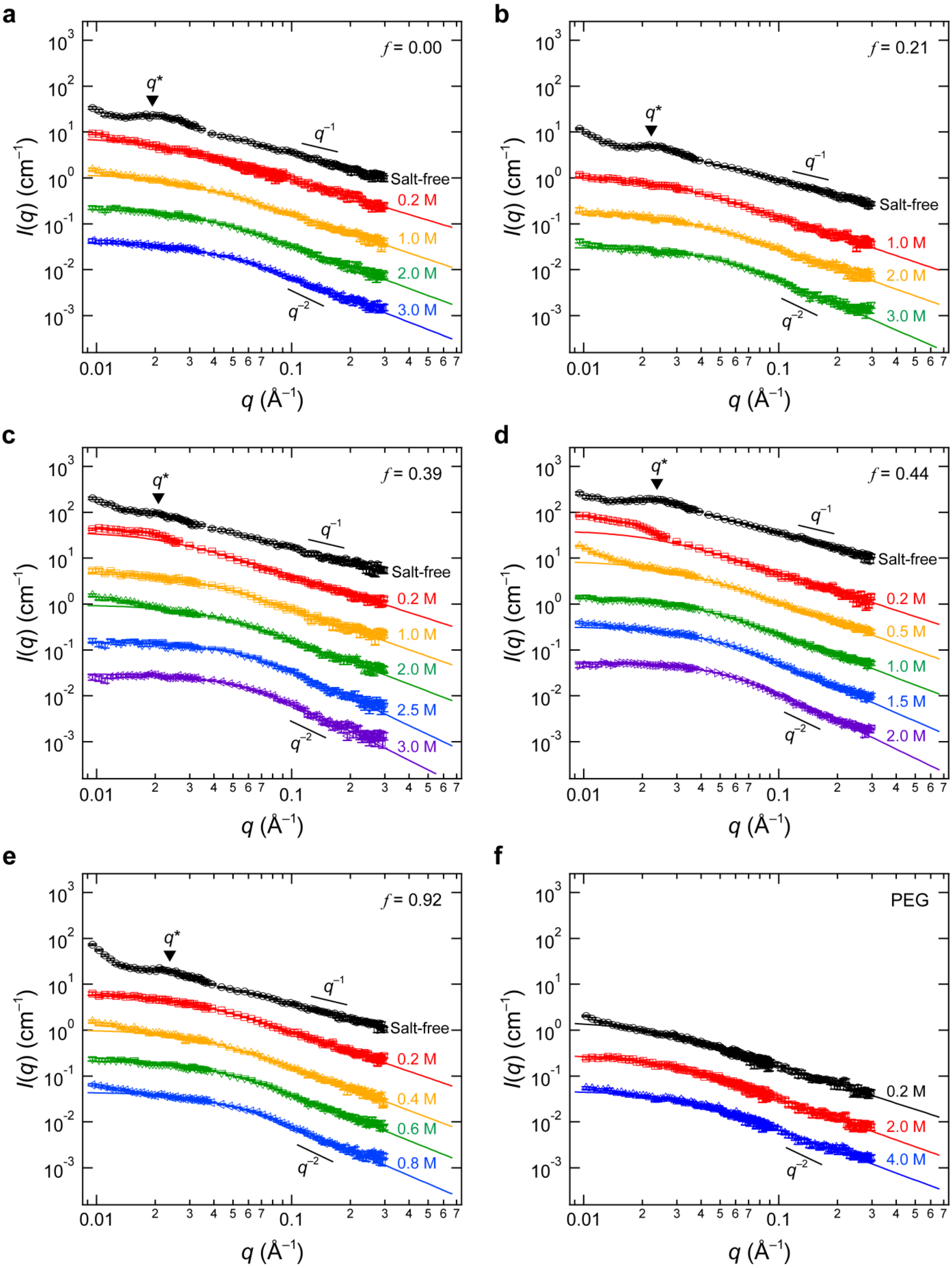

Figure S9. Small-angle neutron scattering (SANS) profiles of poly(G-*ran*-A) solutions with f = (a) 0.00, (b) 0.21, (c) 0.39, (d) 0.44, and (e) 0.92, and (f) of poly(ethylene glycol) (PEG) solutions. The corresponding C_{NaCl} values are indicated in each panel. Solid lines represent the best fit to $P(q)$ according to Equation 2.

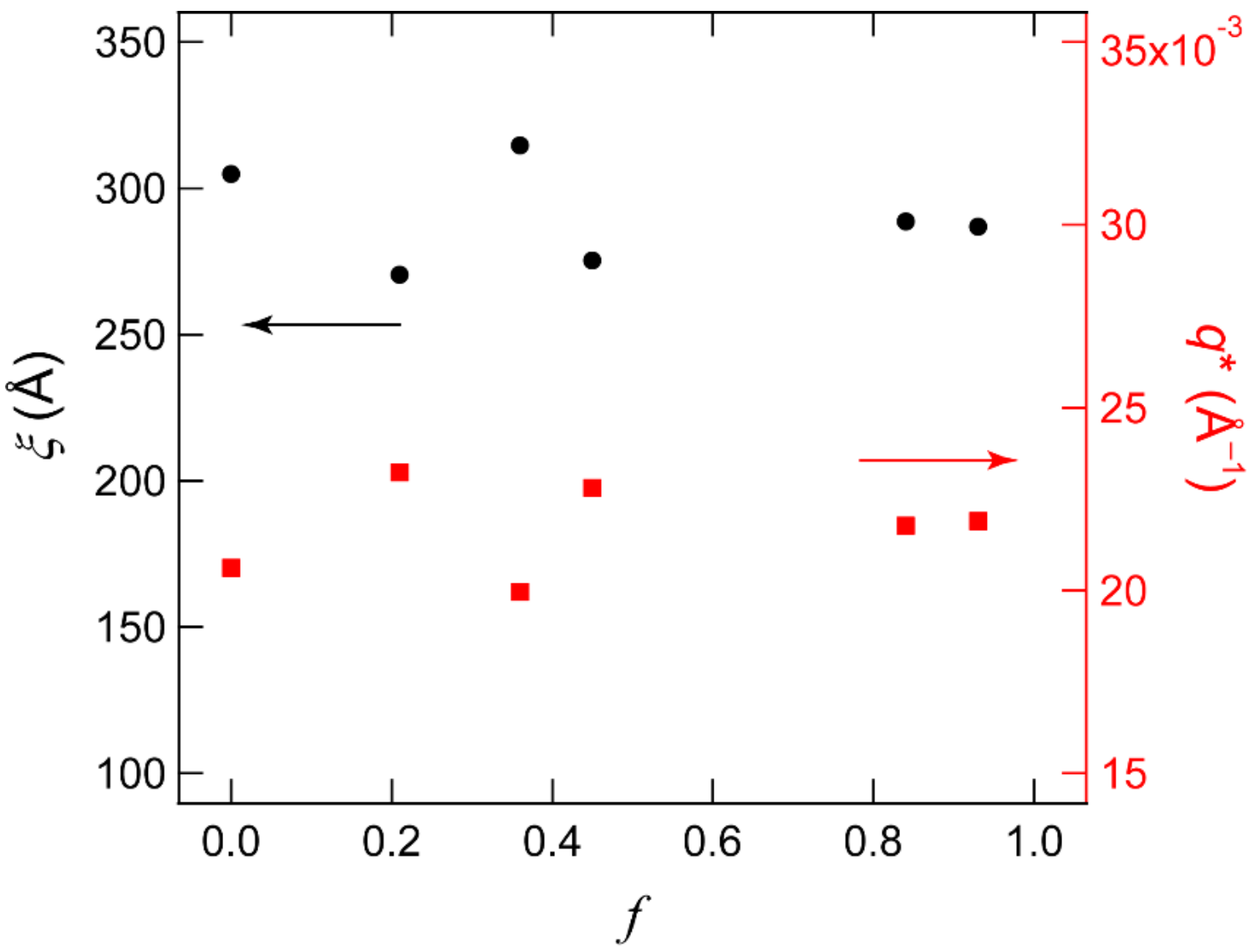

Figure S10. Peak position (q^*) and the corresponding correlation length ($\xi = 2\pi/q^*$) as a function of sticker fraction (f) for salt-free poly(G-*ran*-A) solutions.

4. Static Light Scattering (SLS)

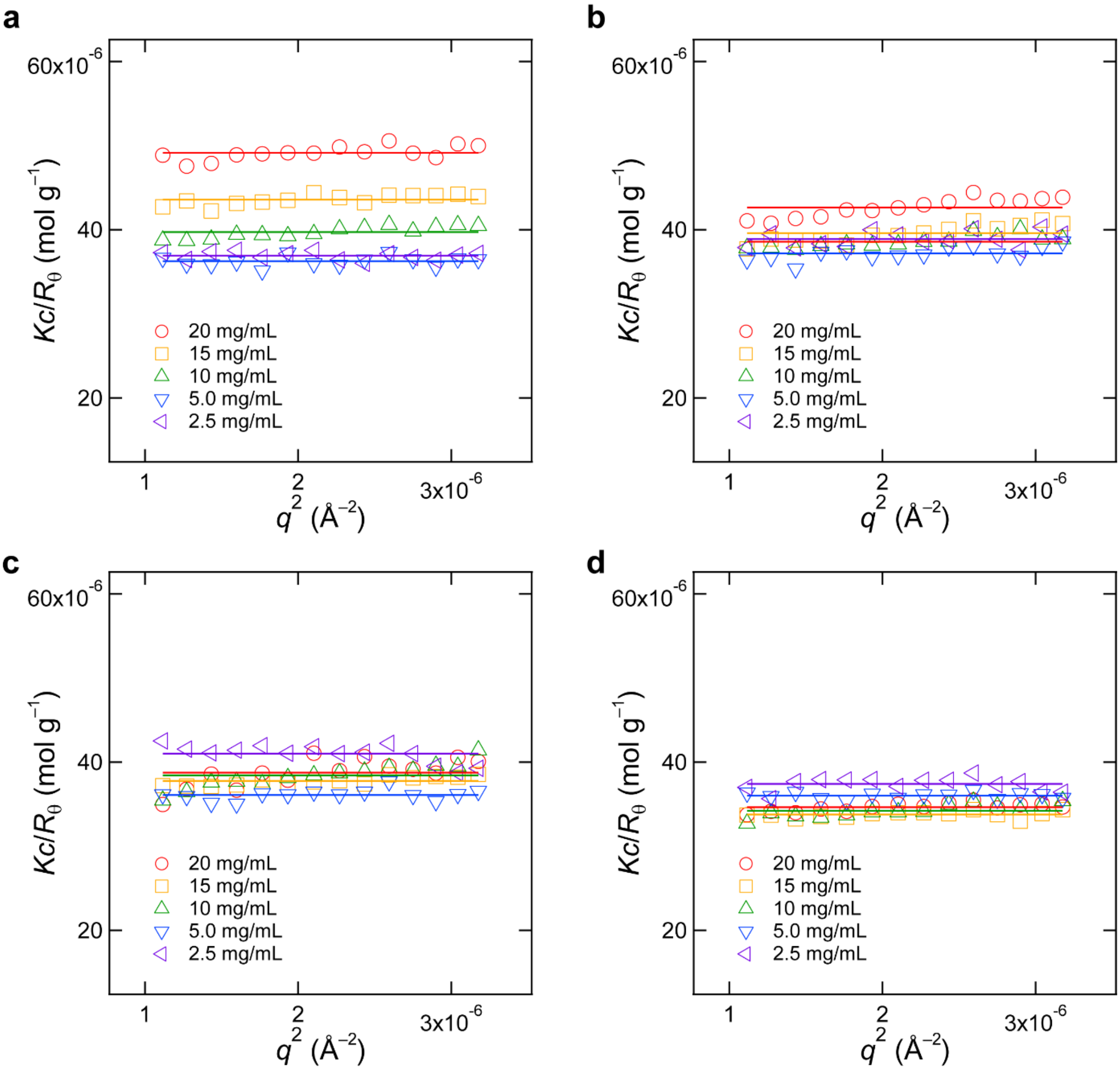

Figure S11. Plots of Kc/R_θ versus q^2 plots for poly(G-*ran*-A) solutions with $f = 0.35$ at C_{NaCl} = (a) 0.5, (b) 1.0, (c) 1.5 and (d) 2.0 M. Solid lines serve as guides to the eyes.

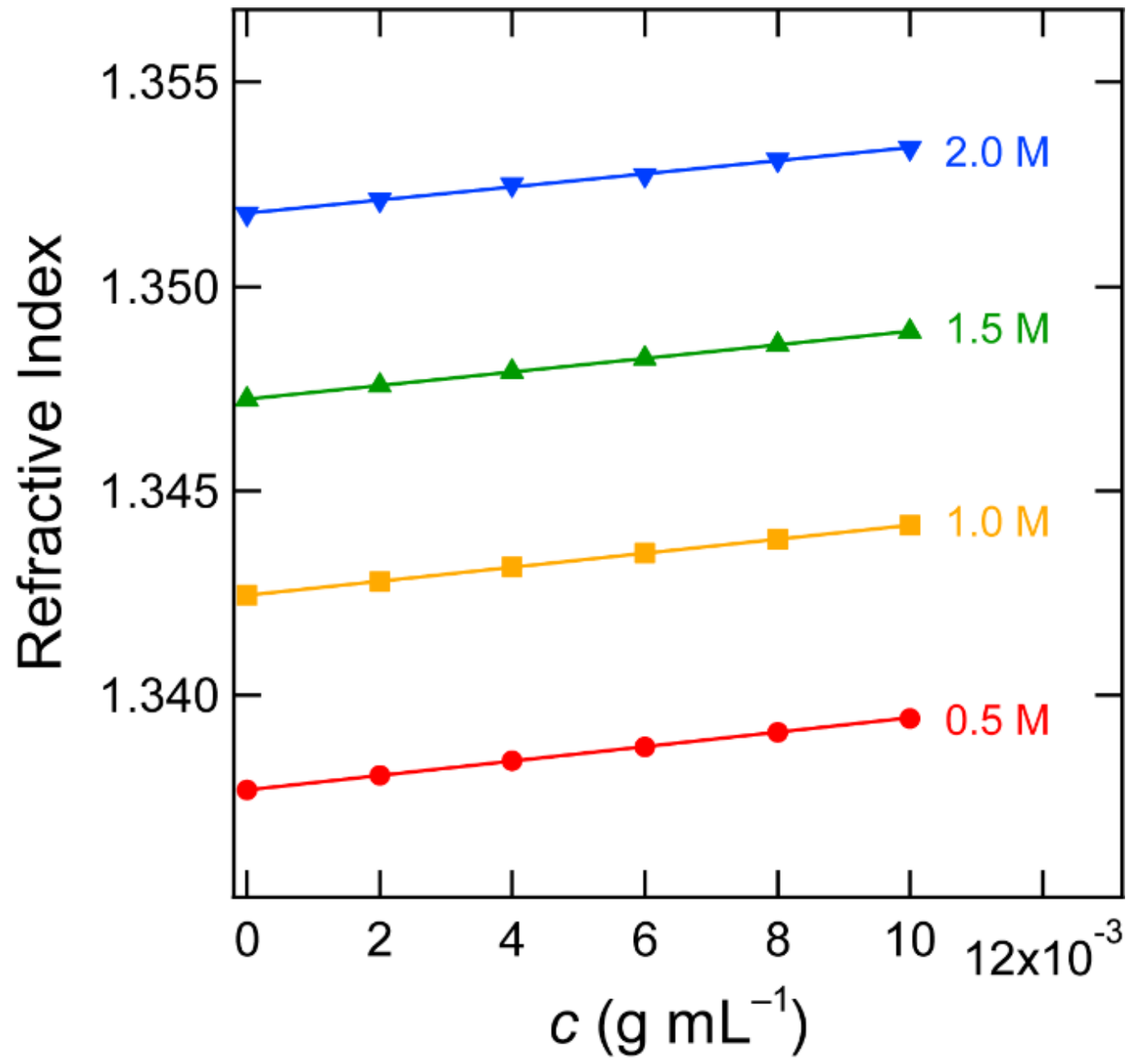

Figure S12. Refractive index (n) of poly(G-*ran*-A) solutions with f = 0.35 as a function of polymer concentration (c) at various C_{NaCl}. The slope yields refractive index increment (dn/dc).

Table S1. Refractive index of solvent (n_0) and refractive index increments (dn/dc) of poly(G-*ran*-A) solutions.

C_{NaCl} (M)	n_0	dn/dc (mL g^{-1})
0.5	1.3377	0.175
1.0	1.3425	0.172
1.5	1.3472	0.166
2.0	1.3518	0.160

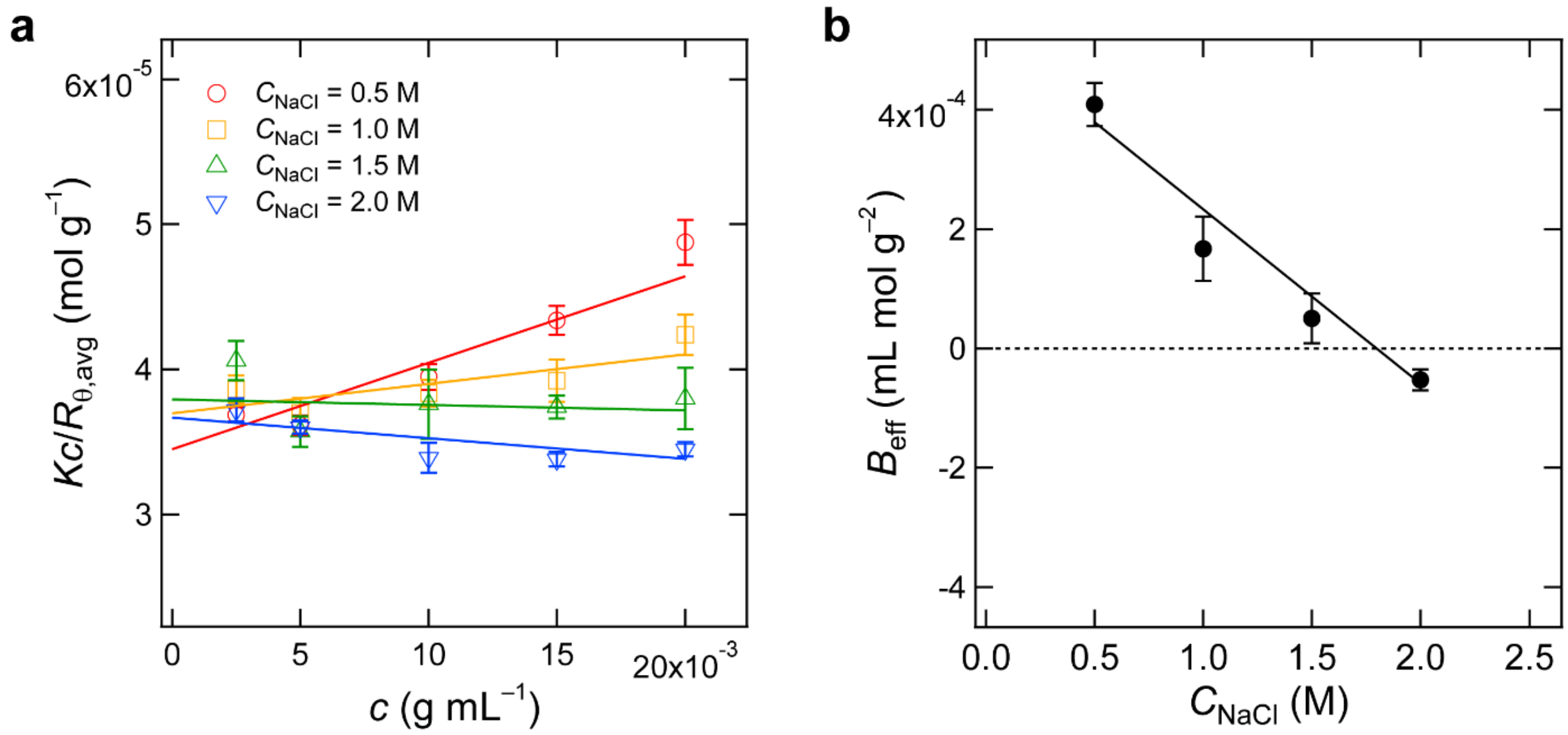

Figure S13. (a) q-averaged Kc/R_θ ($Kc/R_{\theta,avg}$) for poly(G-*ran*-A) with $f = 0.35$ as a function of polymer concentration (c) at various C_{NaCl}. Error bars denote the standard deviation. Solid lines represent the best fit to Equation S5. (b) Effective osmotic second virial coefficient (B_{eff}) of poly(G-*ran*-A) with $f = 0.35$ as a function of C_{NaCl}. Solid line represents the best linear fit. The theta salt concentration (C_θ) is defined as the C_{NaCl} at which $B_{eff} = 0$.

5. Theoretically Informed Coarse-Grained (TICG) Simulations

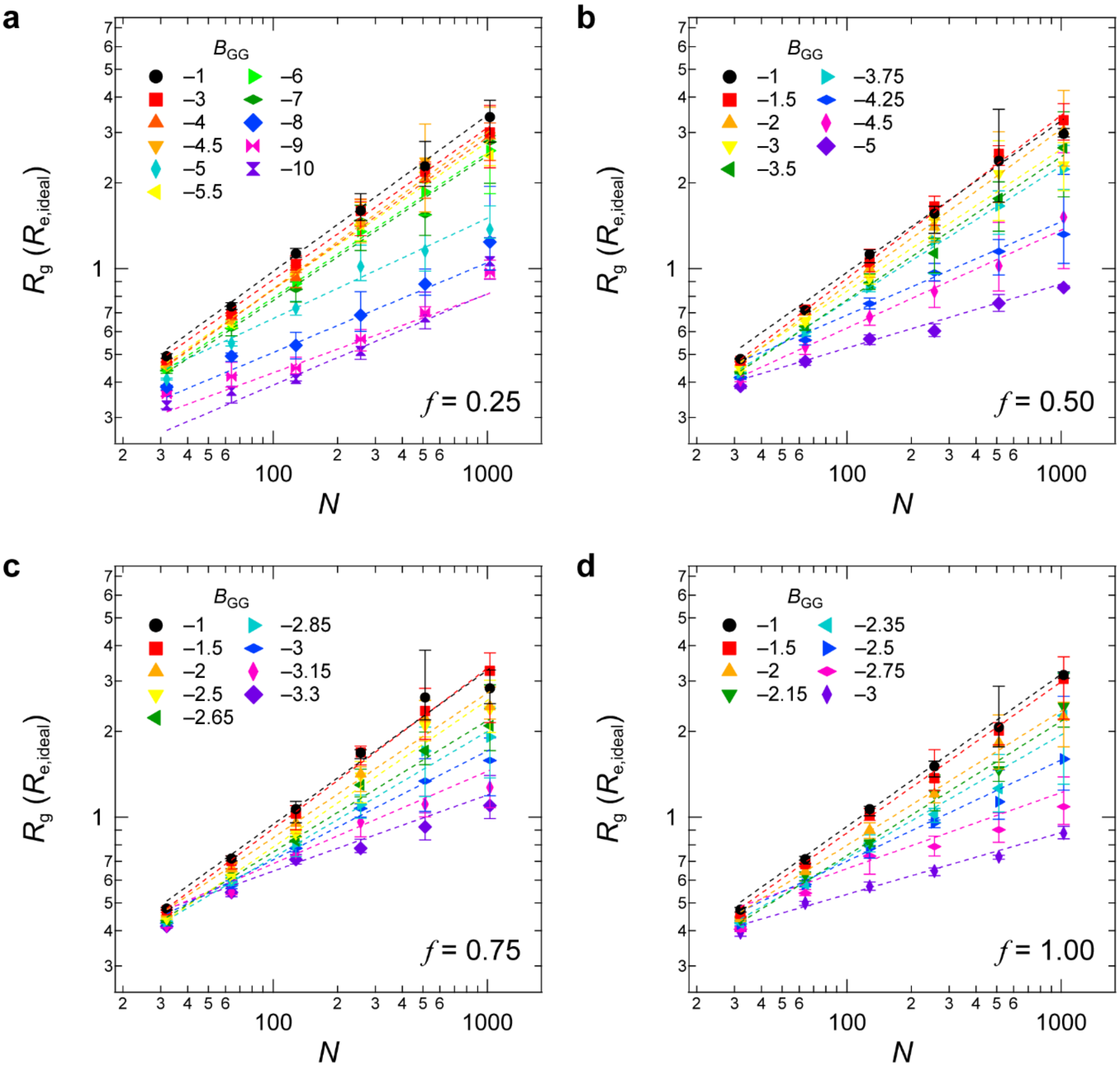

Figure S14. Log-log plots of radius of gyration (R_g), in units of the ideal end-to-end distance under Gaussian conditions ($R_{e,ideal}$), as a function of chain length (N) for coarse-grained poly(G-*ran*-A) chains with f = (a) 0.25, (b) 0.50, (c) 0.75 and (d) 1.00 at various sticker–sticker second virial coefficient (B_{GG}). Dashed lines represent the best pow-law fits used to extract the effective Flory exponent (ν_{eff}).

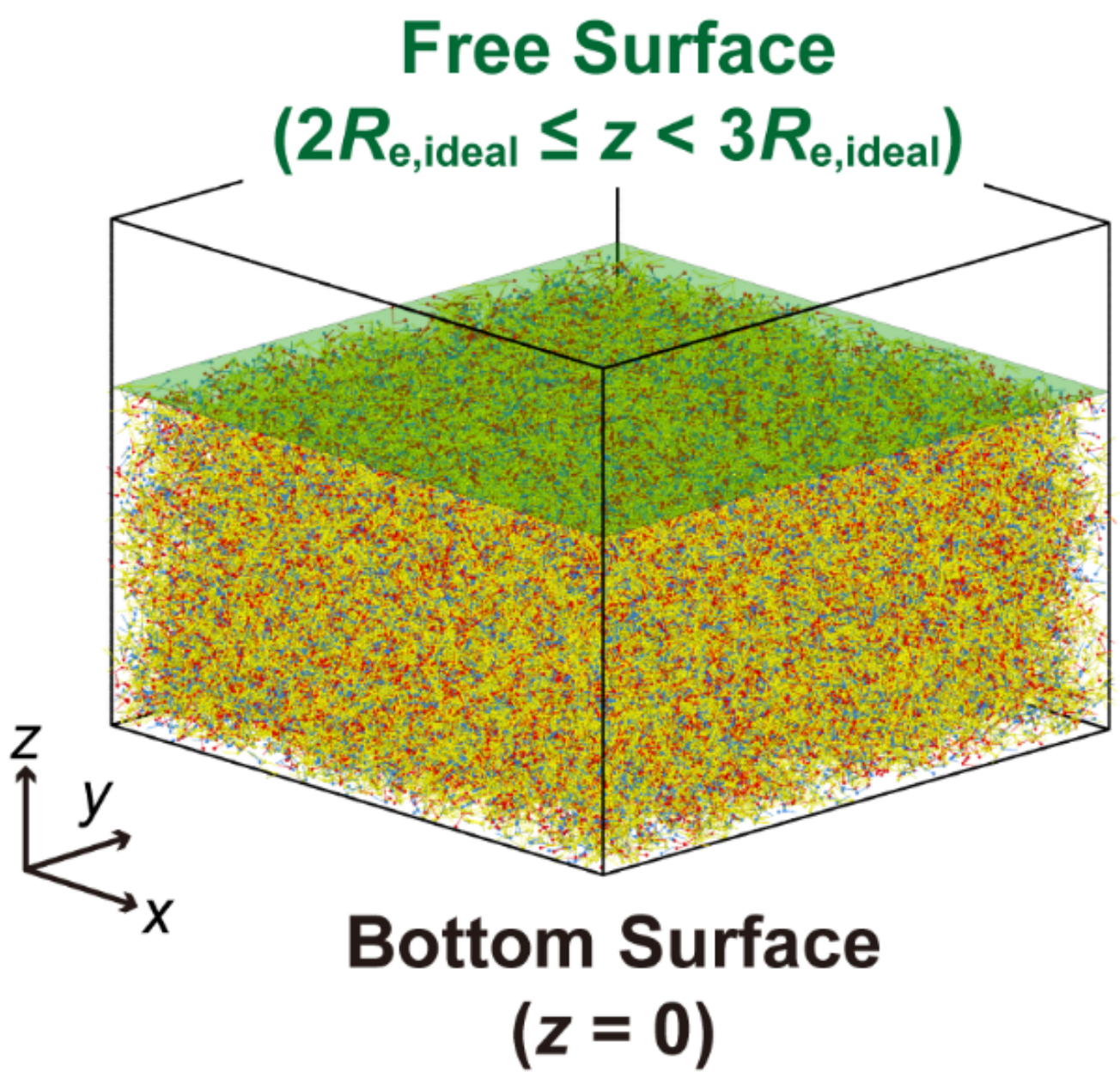

Figure S15. A diagonal-view snapshot of the initial configuration of sticker–spacer polymer films (N = 128, f = 0.50) confined on the bottom surface (z = 0) of the simulation box (4$R_{e,ideal}$ × 4$R_{e,ideal}$ × 3$R_{e,ideal}$). The green shaded area indicates the interface at z = 2$R_{e,ideal}$.

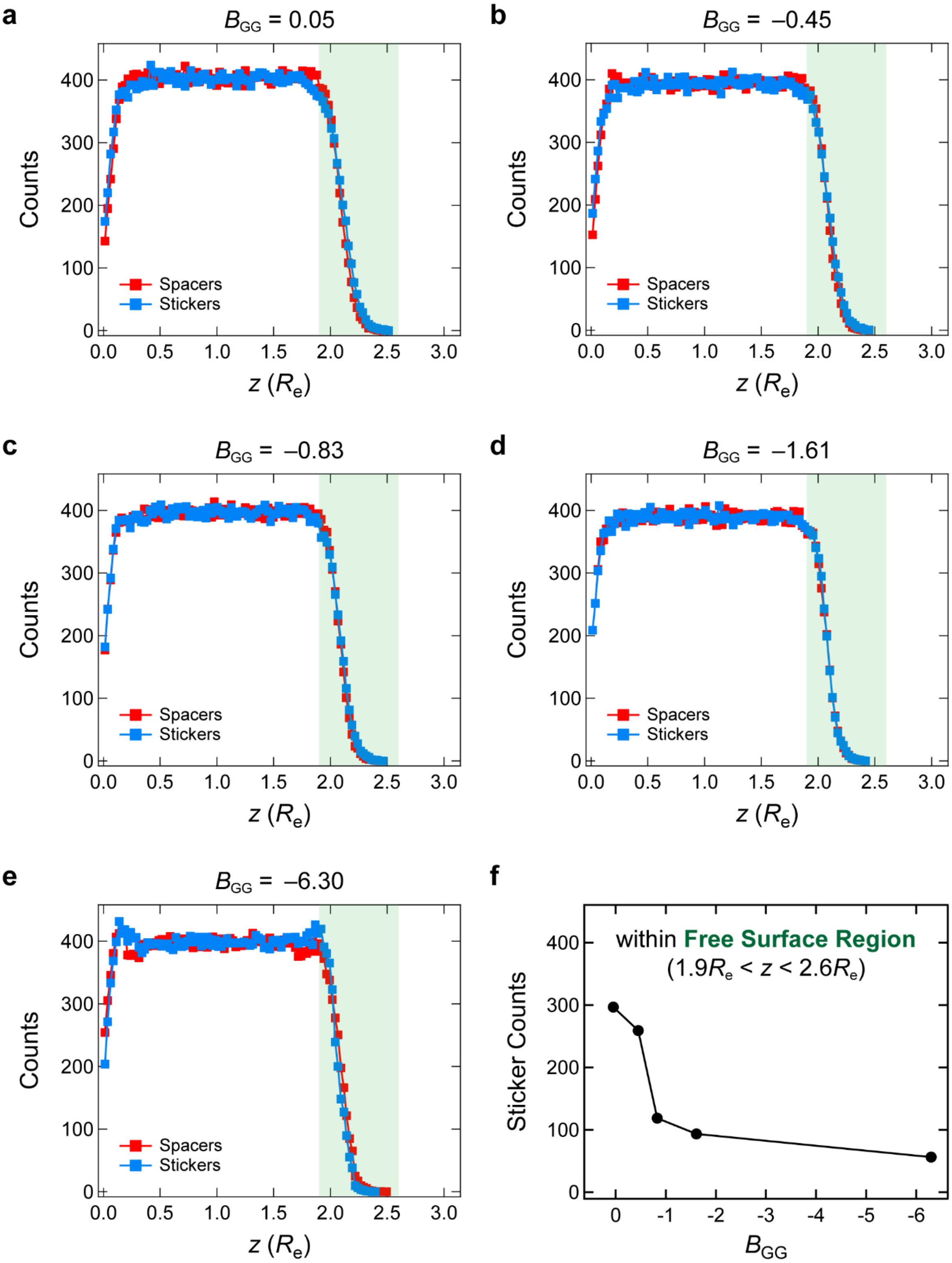

Figure S16. Number distribution of spacer beads (red) and sticker beads (blue) along the z-direction in the polymer-film simulation box at B_{GG} = (a) 0.05, (b) −0.45, (c) −0.83, (d) −1.61, and (e) −6.30. Shaded regions denote the free-surface region ($1.9R_{e,ideal} \leq z < 2.6R_{e,ideal}$). (f) Number of sticker beads within the free surface region as a function of B_{GG}.

Table S2. Second-order virial coefficients (B_{GG}) of sticker beads that have been used in the dilute simulations of single chain sticker-spacer model.

$f = 0.25$	$f = 0.50$	$f = 0.75$	$f = 1.00$
−1.00	−1.00	−1.00	−1.00
−3.00	−1.50	−1.50	−1.50
−4.00	−2.00	−2.00	−2.00
−4.50	−3.00	−2.50	−2.15
−5.00	−3.50	−2.65	−2.35
−5.50	−3.75	−2.85	−2.50
−6.00	−4.25	−3.00	−2.75
−7.00	−4.50	−3.15	−3.00
−8.00	−5.00	−3.30	
−9.00			
−10.00			

Table S3. Second- (B_{GG}) and third-order (ω_{GGG}) virial coefficients of sticker beads that have been used in the film simulations of sticker−spacer model.

B_{GG}	ω_{GGG}
0.04688	0.00018
−0.4475	0.00198
−0.82812	0.00468
−1.60938	0.00933
−6.29688	0.03674

6. Miniature Surface Forces Apparatus (μSFA)

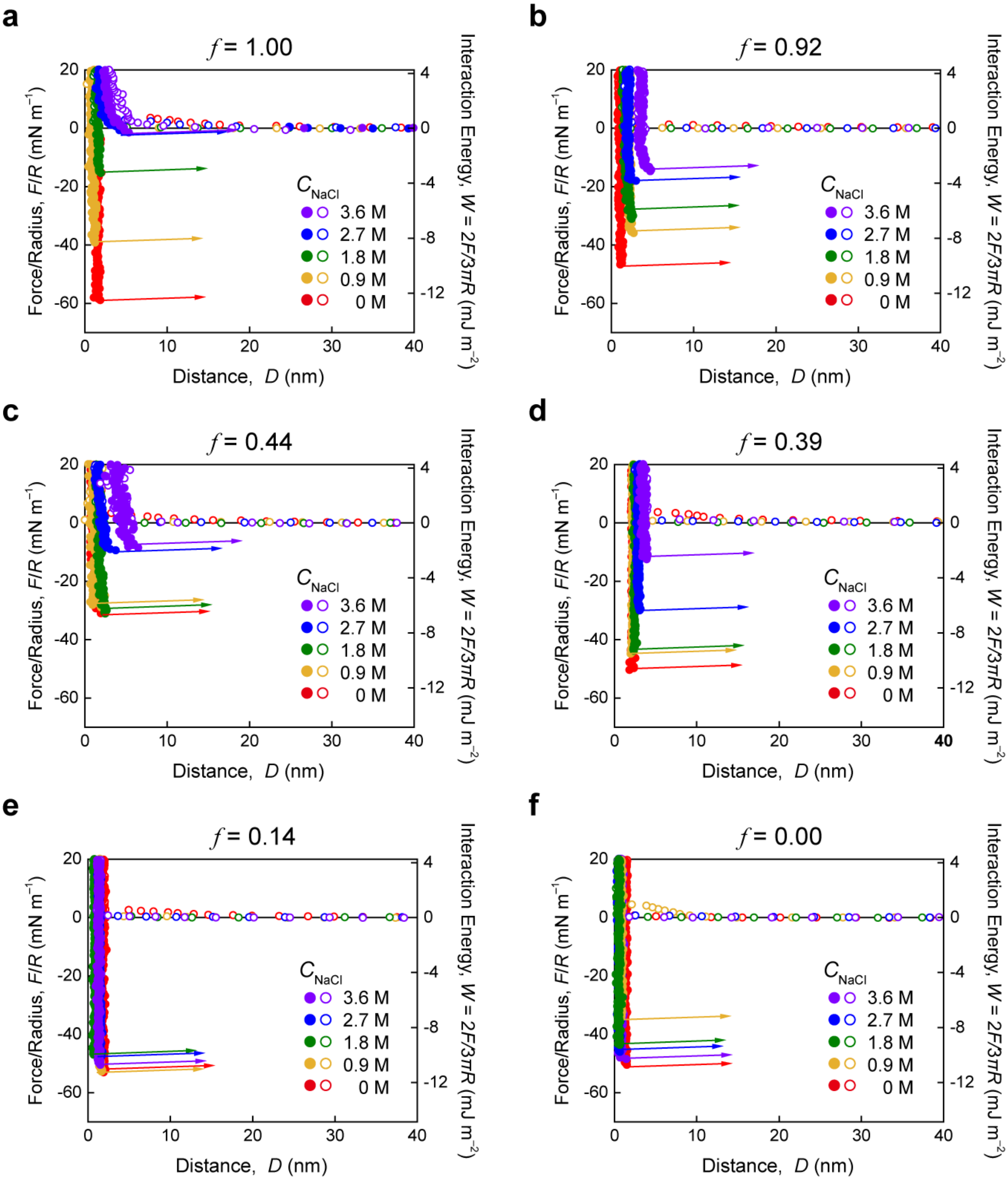

Figure S17. Force–distance profiles recorded during approach and separation of opposing poly(G-*ran*-A) surfaces with f = (a) 1.00, (b) 0.92, (c) 0.44, (d) 0.39, (e) 0.14, and (f) 0.00 at C_{NaCl} = 0 (red), 0.9 (orange), 1.8 (green), 2.7 (blue), and 3.6 M (purple). Filled and open symbols denote the approach and separation process, respectively.

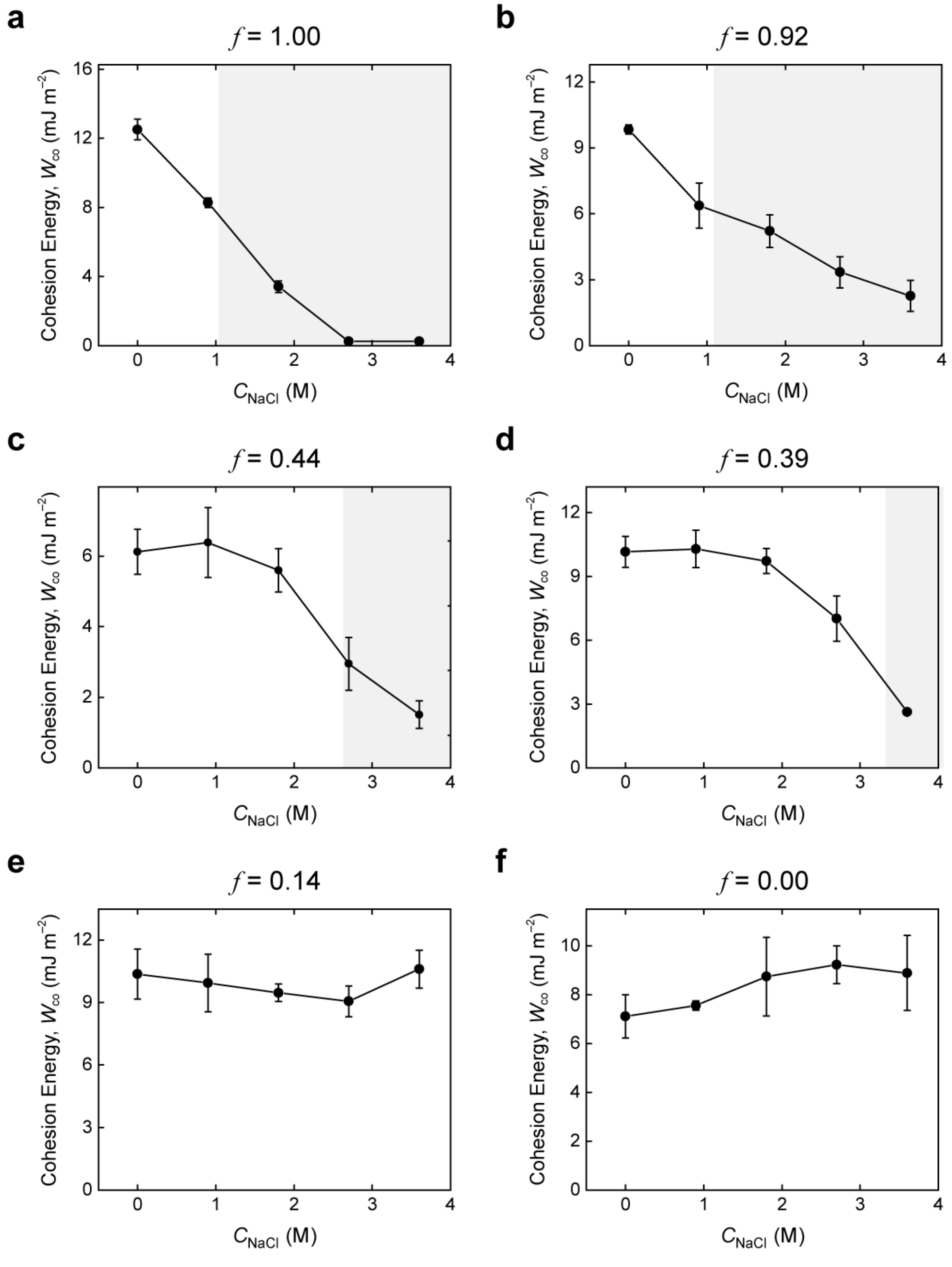

Figure S18. Cohesion energy (W_{co}) of symmetric poly(G-*ran*-A) surfaces with f = (a) 1.00, (b) 0.92, (c) 0.44, (d) 0.39, (e) 0.14, and (f) 0.00 as a function of C_{NaCl}. Error bars represent the standard deviation (n = 5). Shaded regions denote the biphasic phase-separated regimes in dilute solution.

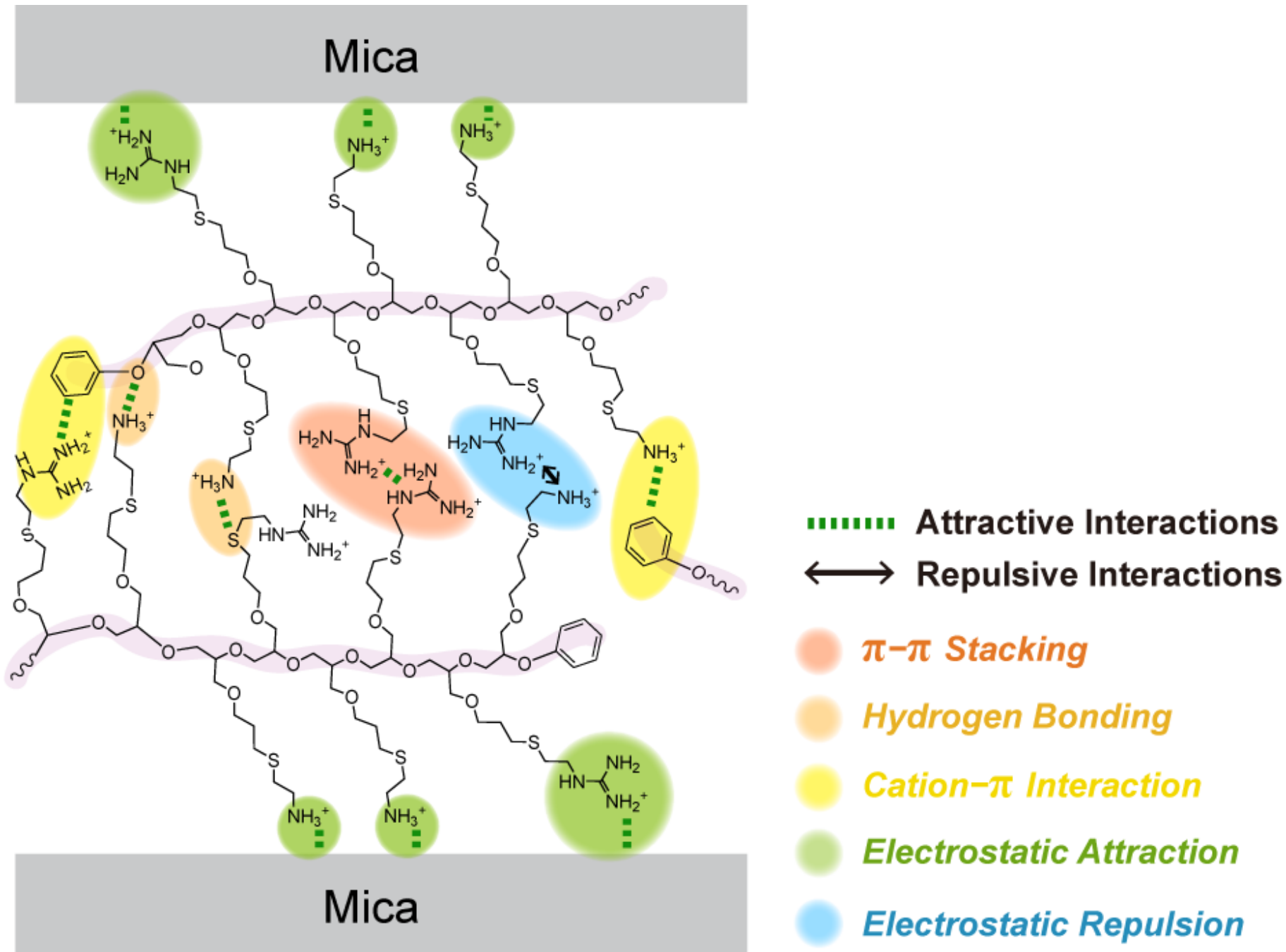

Figure S19. Schematics illustration of possible molecular interactions between symmetric poly(G-*ran*-A) surfaces.

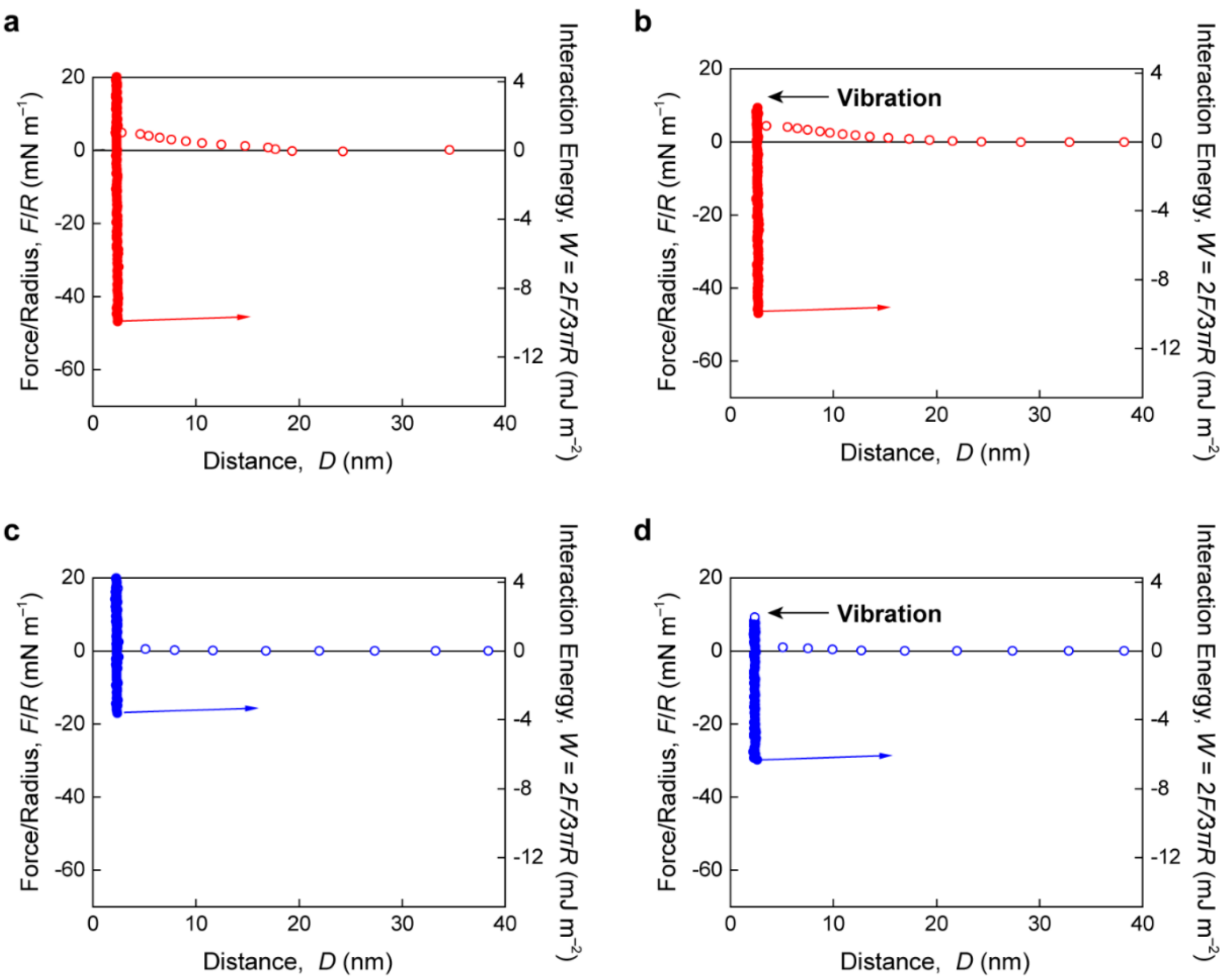

Figure S20. Force-distance profiles during approach and separation of opposing poly(G-*ran*-A) surfaces before and after vibration under salt-free and salt-containing conditions. (a) Before and (b) after vibration in the absence of salt. (c) Before and (d) after vibration at C_{NaCl} = 1.8 M.

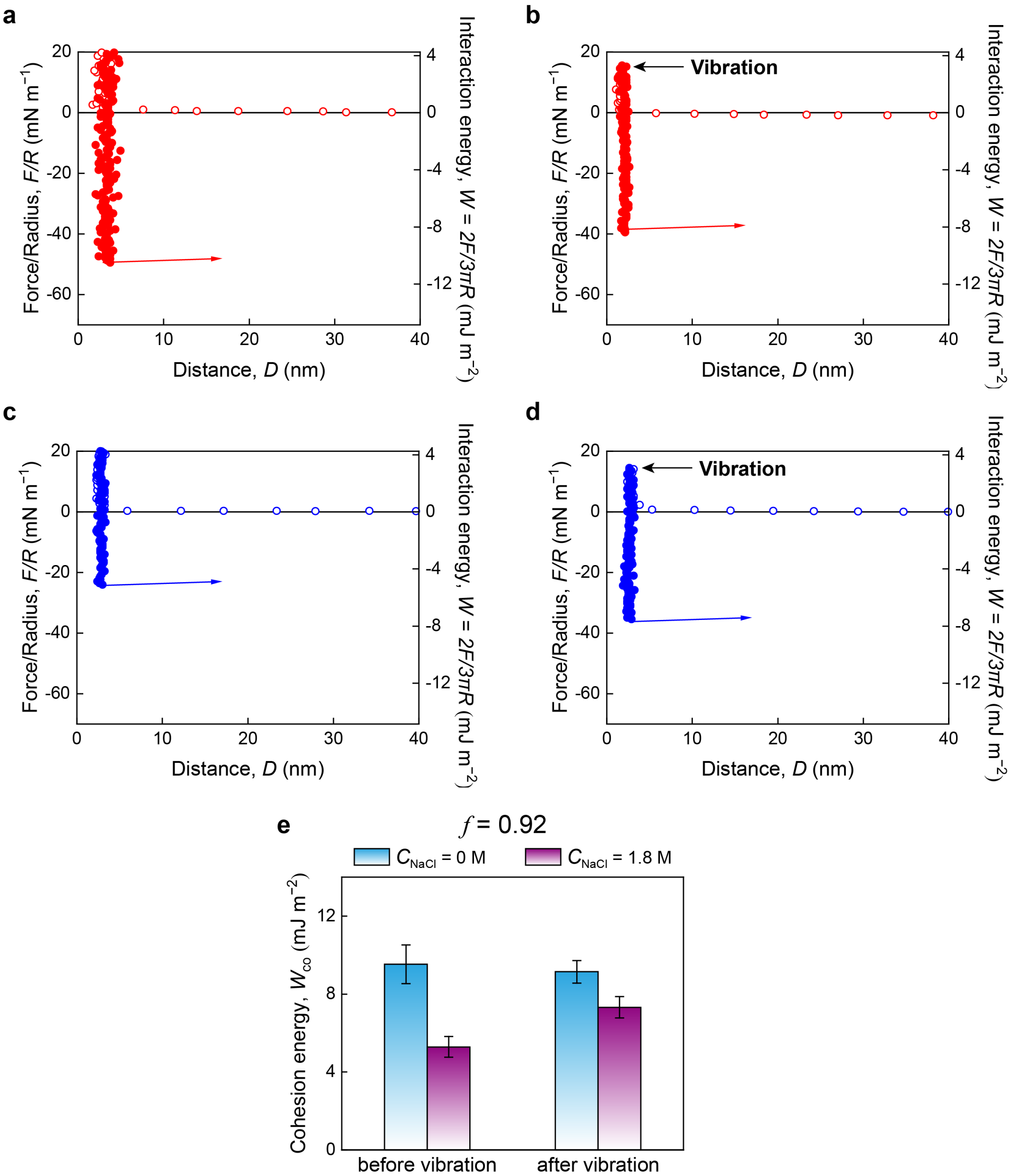

Figure S21. Force-distance profiles during approach and separation of opposing poly(G-*ran*-A) surfaces (f = 0.92) before and after vibration under salt-free and salt-containing conditions. (a) Before and (b) after vibration in the absence of salt. (c) Before and (d) after vibration at C_{NaCl} = 1.8 M. (d) Changes in the W_{co} after applying high-frequency vibration to contacting symmetric poly(G-*ran*-A) surfaces (f = 0.92) at C_{NaCl} = 0 (blue) and 1.8 M (purple). Error bars denote standard deviation (n = 3).

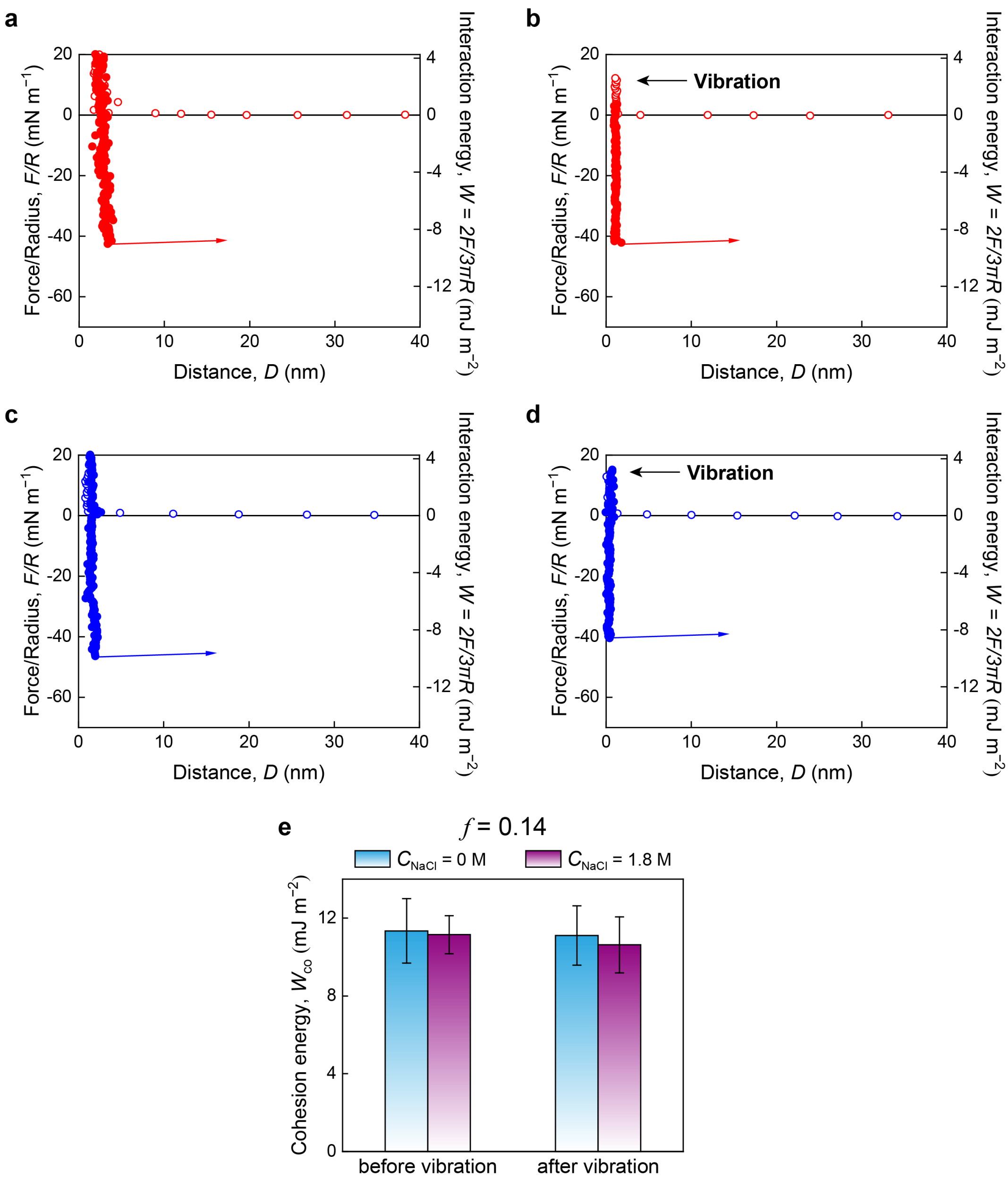

Figure S22. Force-distance profiles during approach and separation of opposing poly(G-*ran*-A) surfaces ($f = 0.14$) before and after vibration under salt-free and salt-containing conditions. (a) Before and (b) after vibration in the absence of salt. (c) Before and (d) after vibration at $C_{NaCl} = 1.8$ M. (d) Changes in the W_{co} after applying high-frequency vibration to contacting symmetric poly(G-*ran*-A) surfaces ($f = 0.14$) at $C_{NaCl} = 0$ (blue) and 1.8 M (purple). Error bars denote standard deviation ($n = 3$).
